# Quality assessment tools used in systematic reviews of in vitro studies: A systematic review

**DOI:** 10.1186/s12874-021-01295-w

**Published:** 2021-05-08

**Authors:** Linh Tran, Dao Ngoc Hien Tam, Abdelrahman Elshafay, Thao Dang, Kenji Hirayama, Nguyen Tien Huy

**Affiliations:** 1 Institute of Fundamental and Applied Sciences, Duy Tan University, Ho Chi Minh City, 700000 Vietnam; 2 Faculty of Natural Sciences, Duy Tan University, Da Nang City, 550000 Vietnam; 3 Asia Shine Trading & Service CO. LTD., Ho Chi Minh City, Vietnam; 4 Online Research Club, Nagasaki, Japan; 5 Faculty of Medicine, Al-Azhar University, Cairo, 11884 Egypt; 6 Department of Internal Medicine, Texas Tech University Health Science Center at the Permian Basin, Odessa, TX USA; 7 Department of Immunogenetics, Institute of Tropical Medicine (NEKKEN), Graduate School of Biomedical Sciences, Nagasaki University, 1-12-4 Sakamoto, Nagasaki, 852-8523 Japan; 8 School of Tropical Medicine and Global Health, Nagasaki University, 1–12–4 Sakamoto, Nagasaki, 852-8523 Japan

**Keywords:** Quality assessment tool, Systematic review, Meta-analysis, In vitro study

## Abstract

**Background:**

Systematic reviews (SRs) and meta-analyses (MAs) are commonly conducted to evaluate and summarize medical literature. This is especially useful in assessing in vitro studies for consistency. Our study aims to systematically review all available quality assessment (QA) tools employed on in vitro SRs/MAs.

**Method:**

A search on four databases, including PubMed, Scopus, Virtual Health Library and Web of Science, was conducted from 2006 to 2020. The available SRs/MAs of in vitro studies were evaluated. DARE tool was applied to assess the risk of bias of included articles. Our protocol was developed and uploaded to ResearchGate in June 2016.

**Results:**

Our findings reported an increasing trend in publication of in vitro SRs/MAs from 2007 to 2020. Among the 244 included SRs/MAs, 126 articles (51.6%) had conducted the QA procedure. Overall, 51 QA tools were identified; 26 of them (51%) were developed by the authors specifically, whereas 25 (49%) were pre-constructed tools. SRs/MAs in dentistry frequently had their own QA tool developed by the authors, while SRs/MAs in other topics applied various QA tools. Many pre-structured tools in these in vitro SRs/MAs were modified from QA tools of in vivo or clinical trials, therefore, they had various criteria.

**Conclusion:**

Many different QA tools currently exist in the literature; however, none cover all critical aspects of in vitro SRs/MAs. There is a need for a comprehensive guideline to ensure the quality of SR/MA due to their precise nature.

**Supplementary Information:**

The online version contains supplementary material available at 10.1186/s12874-021-01295-w.

## Background

Evidence-based medicine (EBM) is a reliable and accurate approach based on existing evidence in healthcare-related researches [1]. Systematic reviews (SRs) and meta-analyses (MAs) are crucial methods of EBM that assess the findings of different work in the medical literature on related topics. The data and conclusions of each work synthesized to present a comprehensive summary and conclusion based on the findings [2, 3]. The primary medical value behind conducting such studies is to improve healthcare delivery and outcomes in the clinical setting. Researchers can utilize these tools to summarize clinical research with a non-biased approach to even the most controversial topics [4, 5]. For in vitro studies, being able to translate and keep track of numerous research projects that address the same topic increases transparency and addresses the significance of clinical translation. They eventually enhance the safety and efficacy of the treatments in clinical practice [6, 7]. Current discrepancy between SRs/MAs on preclinical studies and SRs/MAs on clinical studies suggest a potential gap in the assessment and evaluation of preclinical evidence. This may lead to inadequate translation to clinical evidence [6, 8]. Therefore, further research is justified to address any possible shortfalls in the methodology of performing such study types.

The precise nature of scientific discoveries combined with the increasing influx of research papers highlight the importance of QA tool for all published articles. The development of QA tools investigates to improve the quality of scientific reports by addressing unethical and misconducted research studies [9–11]. There are several methods to assess the quality of SRs/MAs, including Assessment of Multiple Systematic Reviews (AMSTAR) and Critical Appraisal Skills Programme (CASP). The majority of SRs/MAs uses one of many available approaches. However, the content and weights of different tools are variable and inconsistent, which raises the question on either an universally accepted one should be developed. ToxRTool and Oral Health Assessment Tool (OHAT) are two examples of the National Health and Medical Research Council’s recommendation for SRs/MAs of in vitro studies [12]. Both tools cover different aspects of risk of bias, providing researchers with a partial guide while conducting or assessing these studies.

Given the importance of in vitro SRs/MAs to the advancement of research through the comprehensive implementation, its results support the experimental and clinical settings. Further investigations are required to bridge any potential inadequacies in the methods of synthesizing preclinical evidence. The selection of high-quality QA tools for SRs/MAs on preclinical studies can improve research quality by significantly addressing its methodology. Therefore, our study aimed to evaluate all QA tools used in SRs/MAs of in vitro studies.

## Methods

### Protocol registration

Our study followed the steps recommended in the Preferred Reporting Items for Systematic reviews and Meta-Analyses (PRISMA) checklist (Table S1) [13, 14]. The protocol was published in ResearchGate in June 2016 (DOI: 10.13140/RG.2.1.1515.9925). These works relate to in vitro studies; they could not be published on the International Prospective Register of Systematic Reviews (PROSPERO).

### Search strategy

Our conducted search contained two phases. The first phase was performed to identify the SRs/MAs of in vitro studies in October 2016 using four electronic databases, composed of PubMed, Web of Science (ISI), Virtual Health Library (VHL), and Scopus. This search would be later updated in September 2020. The entire search strategy for these databases was provided in Table S2. No restriction was applied inside the publication date range from 2006 to 2020. This procedure was also based on previously performed search [15].

The second phase was conducted to identify potential tools using the Google search engine (www.google.com) [16]. The automatic Google filter was switched off, and the first 300 links of each search term were screened for relevant tools. In addition, the bibliographic references of the first 300 links of Google search and eventually included their respective publications were searched to find additional tools unidentified by the search of the database. The search strategy was described in Table S2. We used this search model as described before in Nolger et al. [17]. Also, several webpages were used to search and identify the relevant QA tools (Table S2).

### Selection criteria

The inclusion criteria of our studies were; 1- The SRs and/or MAs must be purely in vitro research, 2- Search was ranged from 2006 to 2020 for publication year, 3- Original in vitro study was defined as a technique that was conducted in a controlled condition outside the living organism without being implanted again into the living body or organism. The exclusion criteria were; 1- All SRs and/or MAs that involved in vivo studies, 2- Combined in vivo and in vitro studies. After duplicate removal using Endnote X7 program (Thompson Reuter, USA), the titles and abstracts were screened by two reviewers (DNHT and TD) independently, followed by the inclusion and exclusion criteria. Afterward, full-texts of the selected articles were divided into several clusters, and each one was evaluated by another two reviewers (TD and AE) working independently. Results were then gathered, and in the case of inconsistency, a final decision was resolved following discussion with the supervisor (NTH). We included all QA tools that were used in the included articles. For the second phase, all potential QA tools were included if addressed or proposed in any in vitro studies.

### Data extraction

Two independent reviewers (DNHT and TD) extracted the data from included articles into a specifically designed template using the same method in the screening phase. The removed items including the name of authors, year and region of publication, the involvement of a methodologist or statistician, whether a meta-analysis was conducted, and whether the risk of bias assessment was undertaken. To extract the QA tool used in each included study, we followed the name of this tool and searched for its original paper for the subsequent extraction of QA tools. The QA tools included in our study were all tools which the authors applied for included articles in their respective SRs/MAs.

In each confirmed relevant tool, we collected the following components: type of the tool (scale, checklist, or item), number of items and main contents of its tool, the scoring system, description of formulation, whether the tool was developed for generic purpose in SRs/MAs, single-use in a specific SR/MA (in a particular type of in vitro studies), and whether the tools were developed by the authors themselves or pre-structured tools. Reviewers resolved any dissimilarity via discussion. If a decision could not be achieved, the supervisor (NTH) was consulted to reach a consensus.

### Quality assessment (QA)

The QA on the included publications was carried out utilizing the Database of Abstract of Reviews of Effects (DARE) tool [18]. Five criteria consist of: (i) was inclusion/exclusion criteria reported; (ii) was the search adequate; (iii) was the quality of the included studies assessed; (iv) are sufficient details about the individual included studies present and (v) were the included studies synthesized. The interpretation for fulfilling a “yes,” “partial,” and “no” score was described in Figure S1. The DARE tool has been used in a tertiary study to evaluate the included SRs/MAs [19]. Two independent reviewers (LT and TD) performed the QA process, and any dispute was fixed via discussion. If a decision could not be obtained, supervisor (NTH) consulted to reach the consensus. Further analysis was calculated using a Kappa coefficient to determine the inter-agreement between the examiners in each process.

## Results

### Search results

**Fig. 1:**
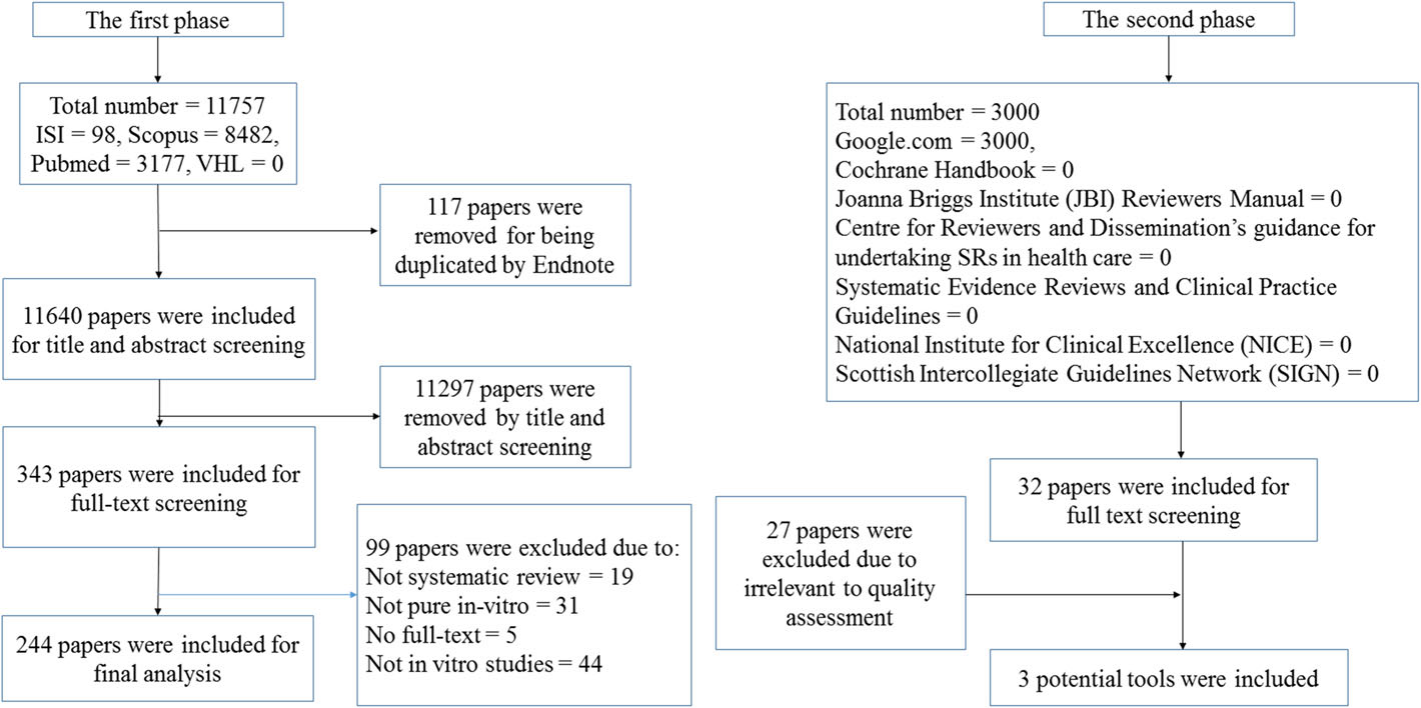
Flow diagram of the search strategy of in vitro SRs/MAs

The first phase retrieved 11,757 initial reports from four electronic databases, including PubMed, ISI, Scopus, and VHL, as shown in Fig. 1. After duplicate removal, the titles and abstracts of 11,640 publications were screened. From this, only 343 studies were included for full-text screening, with 244 articles reaching final eligibility. The list of 99 excluded reports with exclusion reasoning was provided in Table S3. The publication date of all included SRs/MAs ranged from 2007 to 2020. The second phase search retrieved 3000 links in the Google search engine. We screened the first 300 links for each one of ten search terms used. We included 32 links for full-text screening, of which 29 were deemed irrelevant links and were excluded. This left three tools eligible for inclusion in phase two in our study.

### Characteristics of included in vitro SRs/MAs

Among 244 in vitro SRs/MAs included in the analysis, 150 articles (60.7%) employed the guidelines for SRs/MAs. Of these, 146 articles used the PRISMA checklist though only a single study followed Quality of Reporting of Meta-analyses (QUOROM) checklist, and only one study followed Strengthening the Reporting of Observational Studies in Epidemiology (STROBE), and Oral Health Assessment Tool (OHAT) (Table 1). Only 100 articles that followed guidelines for SR/MA reported their QA results. The list of 244 included articles using QA tools in vitro SRs/MAs was found in Table S4.

**Table 1:** Principal characteristics of included articles using QA tools in vitro SRs/MAs

Characteristics	Categorization	All studies (*N* = 244)
Year of publication	2007–2014	24 (9.8%)
	2015–2020	220 (90.2%)
Region	Europe	99 (40.6%)
	South-America	64 (26.2%)
	Asia	33 (13.5%)
	Middle East	26 (10.7%)
	North America	14 (5.7%)
	Australia	5 (2%)
	Africa	3 (1.2%)
Study topic	Dentistry	125 (51.2%)
	Bioactivity	53 (21.7%)
	Biology	31 (12.7%)
	Methodology	13 (5.3%)
	Materials	9 (3.7%)
	Pharmacology	5 (2%)
	Diagnosis	4 (1.6%)
	Toxicity	4 (1.6%)
Reporting QA used	PRISMA	143 (58.6%)
	N	93 (38.1%)
	PRISMA and AMSTAR	3 (1.3%)
	Cochrane Handbook for Systematic Reviews of Interventions	2 (0.8%)
	STROBE	1 (0.4%)
	QUOROM	1 (0.4%)
	OHAT	1 (0.4%)
QA used	Y	126 (51.6%)
	N	118 (48.4%)
Conducting meta-analysis	Y	71 (29.1%)
	N	173 (70.9%)
QA tool used	NR	120 (49.2%)
	Following previous description of Onofre et al.	29 (11.9%)
	Developed by authors	28 (11.5%)
	Cochrane Risk of Bias tool	12 (4.9%)
	CONSORT	8 (3.3%)
	ToxRTool	5 (2%)
	OHAT	4 (1.6%)
	Joanna Briggs Institute Clinical Appraisal Checklist	4 (1.6%)
	MINORS	4 (1.6%)
	QUADAS-2	4 (1.6%)
	GRADE	3 (1.2%)
	NOS	3 (1.2%)
	Following previous description of Onofre et al. and Montagner et al.	2 (0.8%)
	STROBE	2 (0.8%)
	Following the previous description of Bader et al	1 (0.4%)
	Following previous description of Sackett et al	1 (0.4%)
	JADAD	1 (0.4%)
	SciRAP method	1 (0.4%)
	CASP and MINORS	1 (0.4%)
	Timmer’s Analysis Tool	1 (0.4%)
	ARRIVE	1 (0.4%)
	QUADAS	1 (0.4%)
	Modifying Quality Assessment Tool for Studies with Diverse Designs (QATSDD)	1 (0.4%)
	Referencing CRH and the EBM Evidence Pyramid	1 (0.4%)
	Nature Publication Quality Improvement Project (NPQIP) study	1 (0.4%)
	Standard Quality Assessment Criteria for Evaluating Primary Research Papers from a Variety of Fields	1 (0.4%)
	Following previous description of Samuel et al.	1 (0.4%)
	SYCLE	1 (0.4%)
	World Cancer Research Fund/ University of Bristol for cell line	1 (0.4%)
	CRIS guidelines	1 (0.4%)
	Following Joanna Briggs Institute Clinical Appraisal Checklist for Experimental Studies	1 (0.4%)
	PRISMA	1 (0.4%)
	Downs and Black	1 (0.4%)

Abbreviations: N: No; NR: Not Report; Y: Yes; PRIMA: Preferred Reporting Items for Systematic Reviews and Meta-Analyses; AMSTAR: Assessment of Multiple Systematic Reviews; QUOROM: Quality of Reporting of Meta-analyses; QATSDD: Quality Assessment Tool for Studies with Diverse Designs;

The summary statistics are absolute count (%) for categorical variables

Among 244 included studies, 126 articles (51.6%) performed QA. Only 26 of 126 articles developed their QA tools while conducting their reviews, meanwhile 100 articles employed the available tools. Also, 34 studies followed the QA checklist, which was previously developed by other authors. The others assessed the risks of bias following pre-structured QA tools.

Regarding the distribution of the included studies based on the continent, Europe had the most significant representation with 99 (40.7%) studies meanwhile 65 (26.6%), 14 (5.7%), 27 (11.1%), 31 (12.7%) were from South-America, North America, Middle East, and Asia, respectively. Three studies (1.2%) were from Africa, and five studies from (2%) Australia. Table S3 provided the characteristics of all included SRs/MAs.

The publication trend of in vitro SRs/MAs slowly enhanced from 2007 to 2014 and then rapidly increased in the following years until 2020. There were 126 of 244 included articles (51.6%) that conducted methodological QA. Although no SR/MA assessed QA in 2007 and 2008, the prevalent studies performing QA among included in vitro SRs/MAs steadily increased during the search period.

### QA results of included studies using the DARE tool

**Fig. 2:**
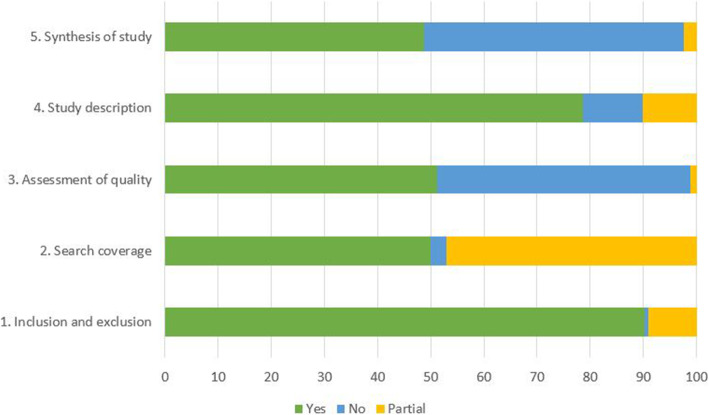
QA results of the included studies using DARE assessment criteria

While utilizing the DARE tool to evaluate 244 included studies, five criteria were presented in Table 2 along with the Kappa’s index and the level of agreement. The inclusion and exclusion criteria have been reported in 220 of included studies (90.2%), while 22 studies were evaluated with partially reporting (9%), and two papers did not report this criterion (0.8%). Search coverage was written in 122 of the included studies (50%), while 115 studies reported partially (47.1%), and seven studies did not report that criterion (2.9%). Among 244 included studies, tools of QA were reported in 126 studies (51.6%); meanwhile, three articles partially performed QA (1.2%), and 115 studies did not assess the QA of their studies (47.2%). Study description and study synthesis criteria have been evaluated, and the level of agreement was critical for both. The QA result of five DARE assessment criteria was provided in Fig. 2 and Table S4.

**Table 2:** Agreement between reviewers of QA of the included studies using DARE assessment criteria

Items	Kappa’ index	Level of Agreement
Inclusion and exclusion	0.94	Almost perfect
Search coverage	0.95	Almost perfect
Assessment of quality	1.00	Almost perfect
Study description	0.89	Strong
Synthesis of study	0.88	Strong

### Summary of QA tools

We identified 51 different available QA tools. Of these, 48 tools from the first phase were retrieved from within included studies and three tools from the second phase found by Google engine, including IVD (in vitro diagnosis), artificial rumen system, and OHAT. We found that 26 used tools (51%) in the first phases developed by the authors [20–45], while other 22 tools were pre-structured and included 19 studies from the first phases and three tools from the second phase accounted for the remaining 49%. Among 26 QA tools developed by the authors, 20 tools (76.9%), specialized in dentistry studies whereas two tools (7.7%) applied in the methodology, two tools (7.7%) applied in bioactivity studies, and two tools (7.7%) involved in the biology studies. Among tools developed by the authors, 17 tools (65.38%) [20, 21, 23, 26–34, 39, 40, 42, 44, 45] mentioned items, which could be used only in specific fields (mainly on dentistry) while nine tools (34.62%) [22, 24, 25, 35–38, 41, 43] contributed the criteria for general reviews, as shown in Table 3. Tools used for a specific study often contained unique factors directly relating to the test materials and outcomes in the reviews. Examples of this include teeth free of caries, the specimen preparation, specimen dimension, enamel antagonist, the specimen shape, concentration of enzymes, storage condition of the sample, or the used devices. The authors also highly concern on the bias of method, which could affect the reliability of outcomes, namely calculating sample size, the randomization of samples, the blinding of the examiner, and the appropriate form of statistical analysis. Instead, tools used for general SRs/MAs evaluated the reliability of methodology to report results generally [43] or consisted of items assessing each step of study (objective, sequence generation, blinding, selection bias, detection bias, performance bias, report bias). The majority of these tools (11 tools, 42.3%) were contributed as simple checklists. These tools only had questions and required the answers of “yes”, “no” or “not report.” The overall bias could be decided by the number of “yes” or “no” answers. Seven checklists with judgment (26.9%) among tools developed by the authors contained multiple items, which required the authors to provide their assessment in details and compared them between studies. Finally, eight scale tools (30.8%) rated the quality of each item with varied levels by giving points to them; for instance, reported answer = 1 point, not reported answer = 0 points. There were also tool in quality ratings of each domain with different levels (0–4 points). The summary score of each study determined as high, low or unclear risk of bias correspondingly.

**Table 3:** Summary results comparing the identified tools by type

Tool characteristics	Developed by the authors (number, %)	Pre-structured tools (number, %)
Purpose		
Items used in specific fields	17, 65.38% [20, 21, 23, 26–34, 39, 40, 42, 44, 45]	5, 20% [46–49]
Items used for general systematic reviews	9, 34.62% [22, 24, 25, 35–38, 41, 43]	20, 80% [50–57]
Characteristics		
Simple checklist	11, 42.3% [22, 24–27, 29, 31, 33, 39, 45]	4, 16% [52, 55, 56]
Checklist with judgment	7, 26.9% [20, 35, 36, 41–43, 45]	7, 28% [46, 47, 49–51, 53, 54]
Scale	8, 30.8% [23, 28, 30, 32, 34, 37, 40, 44]	14, 56% [48, 57]
Total (number, 100%)	26, 100%	25, 100%

In contrast, for 25 pre-structured tools, there were approximately 20 tools (80%) used for general SRs/MAs. The exceptions were QA for IVD [46], a tool for in vitro studies using artificial rumen [47], which specialized in studies on cell lines, and two tools for the evaluation of toxicological/ecotoxicological data [48, 49]. In general, there were four simple checklists, six checklists with judgment with 15 scales respectively (Table 3). IVD and artificial rumen tools are checklists with conclusions. The assessments entirely required the examiners to give their checking based on available criteria. Tool for IVDs suggested validations relating to their technical characteristics, namely technical specification actable for registry purposes, their format for technical file, manufacturers, their proper distribution, and cost-effectiveness.

Similarly, the validation established for experiments with artificial rumen focused on specific criteria via the assessment of microorganisms, dividing protozoa, incubation periods, the digestion, and the interaction between chemicals used. Meanwhile, the tool specializing in cell lines (World Cancer Research Fund, University of Bristol) also highlighted the cell line characteristics, repetitive numbers of experiments, and the reporting selection of outcomes. The first tool in toxicological/ecotoxicological information was developed by Klimisch et al. [49]. The criteria entirely focused on factors affecting the results, namely the test substances (their purity/origin/composition, their concentration/doses) or test systems (their suitability, the physical and chemical characteristics of the medium, negative/positive controls) and method to measure the results (appropriate statistic method). These authors suggested four levels of quality, including reliable with and without restriction, not reliable and not assignable, accordingly. However, this approach did not have specific guidance for the quality evaluation. In 2009, Schneider et al. [48] developed a more detailed tool named ToxRTool based on Klimisch et al. [49] ‘s suggestion to address this flaw. The ToxRTool for in vitro SRs/MAs included 18 questions evaluating the test substances, test system, study design description, study results documentation, and plausibility of study design and data. For each criterion reported, the study gets one point. The summary score will initially determine its level of quality. However, Schneider et al. [48] indicated some critical criteria would downgrade the overall level if the study did not report it. The evaluators will give their decision after considering both the summary score and the answer to critical questions.

For 20 pre-structured tools for general reviews, they emphasized the bias based on the detection or selection of samples, the balance of baseline characteristics, the complete outcome reported, and the sequence generation. Two of these tools (EBM Evidence Pyramid and GRADE tool) were wrongly used as assessment tools of methodological quality or risk of bias. Mainly, Xiao et al. [58] used EBM Evidence Pyramid to evaluate the methodological quality, while Pavan et al. [59] used GRADE tool to assess the risk of bias of their included studies. However, we still had them as exceptional cases of QA tools applied by other authors of SRs/MAs in our research. Among these 20 pre-structured tools, the QA tool referring to CRH and the EBM Evidence Pyramid [50] might be classified as the most straightforward checklist. This tool has four levels and defines the grades of quality based on the study design (SRs/MAs of in vitro studies = A) and baseline characteristics (comparable baseline = B, unknown baseline = C, no similar baseline = D). However, it is inappropriate to evaluate the methodology of a SR/MA only based on the baseline characteristics. The GRADE tool [51] is a tool to grade the quality of evidence (strong to low quality), which consists of six other domains (study design, inconsistency, indirectness, limitations, imprecision, and publication bias) to adjust (downward or upward) this initial assessment of quality. Therefore, the GRADE tool instructs the authors on defining the critical outcomes and evaluating the quality of such results rather than assessing the study’s risk of bias.

For the remained 18 tools, although there were both checklists with available questions needing yes/no answers and lists with domains needs requiring assessor’s opinions, these questions are divided into these domains: rationale of the study, samples, randomization, blinding, procedures, reported outcomes, discussion evaluation and other bias (Table 4). The criteria were highly varied. The most popular criterion, which needs to be considered as the appropriate analysis, was mentioned in Cochrane Collaboration [60], Joanna Briggs Institute Clinical Appraisal Checklist for Experimental Studies [61], Timmer’s Analysis Tool [52], and OHAT [53]. Other pricipal criteria are description of data collection, the blinding of samples and investigators/assessors, appropriate method, reporting of all outcomes mentioned in the method, and reporting of missing data. These criteria were mentioned by three tools in Table 4. The less highlighted criteria include the reasonable sample size, the appropriate method of data collection, the representative samples, the balanced baseline characteristic between intervention groups, the detailed sample data, the randomization of allocation sequence, the assurance that samples received the proper procedure, the appropriate control/reference standards, and the adjusted confounders. Finally, the criteria rated by only one tool are the rationale of the study, the description of the sample collection tool, the description of controls/reference standards, the adequate randomization, the blinding of allocation sequence, the full description of procedures, the identical approach between groups, the description of control/reference standard, the replication, the justification of method analysis, the similar research between groups, the report of complete data, no selection of reported results, report of intermediate results and the requirement of the reflection in a clinical trial.

**Table 4:** The criteria rated by five tools (Cochrane collaboration, Joanna Briggs Institute Clinical appraisal checklist for experimental studies, QUADAS tool, Timmer’s analysis tool, OHAT)

Criteria		Cochrane Collaboration	Joanna Briggs Institute Clinical Appraisal Checklist for Experimental Studies	QUADAS tool	Timmer’s Analysis Tool	OHAT
Rationale of study	Rationale of study	–	+	–	–	–
Sample						
	Reasonable sample size	–	+		+	–
	Description of data collection	–	+	+	+	–
	Appropriate method of data collection	–	+	–	+	–
	Sample collection tool	–	+	–	–	–
	Representative/ appropriate samples	–	–	+	+	–
	The balanced baseline characteristics between intervention groups	+	–	–	–	+
	Detailed sample data	–	+	–	+	–
	Description of control/reference standard	–	–	+	–	–
	Appropriate control/reference	–	–	–	+	–
Randomization						
	Randomization of allocation sequence	+	–	–	+	–
	Adequate randomization	–	–	–	–	+
Blinding						
	Allocation sequence	+	–	–	–	–
	Sample/Participants	+	–	–	+	+
	Investigators/Assessors	+	–	–	+	+
Procedure						
	Full description of procedures	–	+	–	–	–
	Samples received proper procedure	+	–	+	–	–
	Identical procedure between group	–	–	–	–	+
	Choice of appropriate method	–	–	+	+	+
	Appropriate control/reference standard	–	–	+	–	–
	The ability for replication	–	–	+	–	–
	Appropriate analysis	+	+		+	+
	Justification of method analysis		+	–	–	–
	Identical analysis between group	+	–	–	–	–
	Adjust confounders	–	–	–	+	+
Reporting outcomes						
	Complete reported results	+	–	–	+	+
	Complete data	+	–	–	–	–
	No selection of reported results	+	–	–	–	–
	Intermediate results reported	–	–	+	–	–
	Missing data reported	+	–	+	+	–
	Clinical practice reflection	–	–	+	–	–

+ This is a criterion of the tool

- This is not required

## Discussion

The publication trend of in vitro SRs/MAs has been recently increasing [62]. This was demonstrated by the number of SRs/MAs found in our study along with the recent surge of novel QA tools for in vitro studies [48, 53]. QA highly plays a critical role in every SR/MA to judge the methodology and reliability, reduce the risk of bias, and strengthen the evidence and recommendations taken from such reviews [63]. However, the percentage of papers, which reported QA procedures in our SRs/MAs, was associated with an increasing trend to 42% in 2016. This was relatively low compared to other areas, such as in randomized clinical trials, the Cochrane tool was reported as widely used in 100% Cochrane reviews [64].

In our study, a total of 51 tools has been identified from two phases, in which, 48 tools were used by authors of the included studies and three tools were found via Google engine. There were 26 articles, which used in the authors’ methodological assessment, and other tools were pre-structured. Almost every study used a different QA tool, which imposed several challenges that might restrict the process of consistent, reliable, and integral appraisal of SRs/MAs. Most QA tools in SRs/MAs of dentistry topic were developed by the authors. These tools were mainly methodological QA that primarily focused on materials and standard procedures in dentistry. Also, the majority of dentistry SRs/MAs followed by the criteria previously proposed by Onofre et al. This implied that the criteria for assessing the methodology in dental procedures were standards and could be applied widely in different SRs/MAs. Only few dental SRs/MAs followed QA process as the instructed in pre-structured tools such as Cochrane or CASP and MINORS [65–69].

Regarding SRs/MAs relating to toxicology and diagnosis, there was a consistent manner among applied tools since these QA tools specialized in each specific topic. For instance, ToxRTool was applied in toxicological studies and QUADAS-2 used in diagnosis studies. We also recommend these tools in SRs/MAs of these particular topics because the criteria mainly cover the essential aspects from the included studies. Other SRs/MAs of other subjects (biology, bioactivity, and materials) assessed the quality of included studies primarily by pre-structured tools. However, they considered a variety of selected QA tools used. Both methodological and reporting QA tools were applied. The authors had to modify these tools for their in vitro SRs/MAs since these tools were initially applied for in vivo studies (SYCLE tool) or clinical trials (MINORS, CONSORT checklist, Cochrane risk of bias tool). This resulted in several inappropriate criteria, which were only applicable in clinical trials or in vivo studies, which were included in assessing quality. Only few SRs/MAs developed their own criteria for these assessments [23, 70, 71]. As a result, this variety of QA tools poses a difficulty for researchers to select which tool is more suitable to apply. Also, the inconsistency of items covered by each tool will affect the review process, especially when a specific tool dismisses items that reflect the essential informations on the study under-assessment. This mostly occurred in methodological QA tools since no current tool covers all methodological aspects for all topics. Therefore, this emphasizes the importance of reaching a consensus among researchers regarding QA tools for in vitro SRs/MAs comprehensively. Possibly there were several inappropriate criteria included in a tool and lacking of several critical criteria, there is a need for consensus of essential aspects that should be included in QA tool of in vitro SRs/MAs as well as the removal of unnecessary criteria.

Given the lack of a standard QA tool for in vitro studies, several authors tend to develop a checklist that suits the needs of their project. Passos et al. [20], Altmann et al. [21], and Goldbach et al. [25] have developed different sets of QA tools that are either generic or serve specific purposes. Sarkis-Onofre et al. [26, 72–77] also developed several scales for single use in a particular context. This tool was used by other in vitro studies of the same topic in our sample and consisted of seven items. The most commonly used items among seven studies including sample size calculation and randomization of ceramic specimen/teeth, indicated a specific field of in vitro research. A highlight of tool developed by authors in conducting their study is their technical characteristics. These assessment help the authors evaluate the exact level of quality of included studies by relevant factors that affect the reliability of these studies (including the used devices, the appropriate medium, or specimens). However, the tools missed non-technical factors causing bias, such as the integrity of data reported or the appropriate analysis method. Three other tools [22, 24, 25], which were developed by authors but covered different aspects of a study, appeared to comprehensively evaluate their included studies ranging from report/performance/selection/detection criteria. However, the guidance for using these tools was not clarified in these reviews, as they only rated these criteria based on their own research question leading to the limitations if other reviews aimed to use their approaches. The specificity of these tools limits their use on a broader scale, and the variant nature facilitates a researcher to disregard specific tools in literature searching.

ToxRTool, which was known as criteria for reporting and evaluating ecotoxicity data, and other standardized tools were developed to provide more detailed and transparent evaluation systems [78–80]. ToxRTool has become widely used in toxicological research, and there are similar tools adapted from it [81]. However, the consistency of ToxRTool has some limitations and requires some refinements [82]. In fact, ToxRTool aimed to evaluate the toxicological data. Therefore, there are many questions relating to the test substance and test systems that might not be important for other in vitro studies, for instance, the pureness and source of the test substance.

Moreover, the lack of positive control would reduce points of the quality of a study. However, not all in vitro studies have their positive control. This decision depends on the research questions and the purposes of the study. Several other crucial factors for general in vitro studies were neglected in this tool, such as the description of sample collection or the suitable sample size. For these reasons, ToxRTool might be the best choice for toxicological studies. Nevertheless, if we used it for reviews of in vitro studies in other fields, it could not assess the appropriate levels of quality for included studies.

Amongst pre-structured tools for general reviews included in our study, EBM Evidence Pyramid and GRADE tool were wrongly applied to assess the methodology and risk of bias. We do not recommend other authors to use these tools in their SRs/MAs in the future. On the other hand, it seems that no tool covers almost the essential criteria, such as sample size, the procedure of sample collection, negative/positive controls, randomization, blinding, analysis, data complete, and missing data. For instance, the justification of sample size, the full description of experimental procedures, and the appropriate positive/negative controls were only included for assessment in limited tools.

Similarly, the report of missing data and the completion of data report are also important since they relate to the reporting bias leading to the inaccurate reflection of in vitro results in clinical trials. But these criteria are only mentioned in one tool. In contrast, for in vitro studies, the blinding of the sample (participant) is unnecessary since this kind of sample could not cause report bias. However, this criterion is requested to report in many tools due to the modification of QA tools of in vivo studies or clinical trials. This gap leads to the fact that whether the authors use any tools for their reviews, the assessment might not reflect the proper level of quality of included studies. This depends on authors’ perspective to determine which tool is more relevant to their research questions. Frequently, the authors should combine several tools to get the most suitable one for their study. But this could not be applied widely if no detailed guidance is developed. Three tools mentioned the randomization, in which, Cochrane Collaboration and Timmer’s Analysis Tool suggested the randomization of allocation sequence meanwhile OHAT recomended assessing whether the randomization was adequate. This is a challenge for in vitro reviews since the randomization is difficult to apply for in vitro materials. Unfortunately, no in vitro review in our study clarified how they determined the randomization in their included studies. We do not deny the critical role of randomization to reduce the selection bias and reduce the wrong results caused by different characteristics of samples. However, we suggest that additional QA tools for in vitro studies should focus on criteria to assure the identical attributes in studied samples. This finding is easier than the contribution to the method of randomization. Finally, we agree that a QA tool for in vitro studies should include the blinding of investigators and assessors. This can reduce the bias caused by the ability to predict the results of the researchers.

Our study had certain limitations. Despite low probability, we applied the restrictions to our search during 14 years, excluding in vivo studies and combined studies. In addition, 300 links screening from Google search engine might result in a missed tool or guideline. Also, we included all tools applied by the authors in their SRs/MAs that led to forming some inappropriate QA tools (GRADE tool and EBM Evidence Pyramid). However, we discussed this problem and did not recommend it in other SRs/MAs of in vitro studies.

## Conclusions

Multiple different QA tools are currently available throughout the literature. However, none could cover all critical aspects of in vitro SRs/MAs. Thus, a comprehensive guide should be developed to addresses all significant concerns and aspects of this field. This would have the possibility to increase the transparency and reproducibility of scientific work, boosting the reliability and validity of available in vitro findings. This study serves as an initial step towards achieving these targets by summarizing the QA tools that are readily utilized throughout the literature while pointing out potential improvements to be adopted in the future.

## Supplementary Information

**Additional file 1: Table S1.** PRISMA checklist.

**Additional file 2: Table S2.** Detailed search strategy for each database search.

**Additional file 3: Table S3.** List of excluded studies.

**Additional file 4: Table S4.** List of included articles using QA tools in in vitro SRs/MAs.

**Additional file 5: Table S5.** Detailed result of DARE assessment criteria of included studies.

**Additional file 6: Figure S1.** The interpretation for fulfilling a “yes”, “partial” and “no” score.

## Data Availability

The corresponding authors will provide the datasets in this study by reasonable request.

## Abbreviations

AMSTAR: Assessment of Multiple Systematic Reviews
CASP: Critical Appraisal Skills Programme
CRD: Center for Reviews and Dissemination
DARE: Database of Abstract of Reviews of Effects
EBM: Evidence-based medicine
IVD: In vitro diagnosis
JBI: Joanna Briggs Institute
MA: Meta-analyses
NICE: National Institute for Clinical Excellence
PRISMA: Preferred reporting items for systematic reviews and meta-analyses
QA: Quality assessment
QUOROM: Quality of Reporting of Meta-analyses
SIGN: Scottish Intercollegiate Guidelines Network
SR: Systematic reviews
VHL: Virtual Health Library
