# Supplementary material for: Quality assessment tools used in systematic reviews of in vitro studies: A systematic review
Source: BMC Med Res Methodol. 2021 May 8;21:101. doi: 10.1186/s12874-021-01295-w (PMC8106836; doi:10.1186/s12874-021-01295-w)
Supplement: Supplementary file 2 — Additional file 2: Table S2. Detailed search strategy for each database search. [file 12874_2021_1295_MOESM2_ESM.docx]

| ***No.*** | ***Databases*** | ***Search Terms*** | ***Results*** |
| --- | --- | --- | --- |
| 1 | PubMed | "*in vitro*"[All Fields] AND ("systematic review"[All fields] OR "meta-analysis"[All fields] OR "meta-analysis"[Publication Type] OR ("Cochrane Database Syst Rev"[Journal] OR ("Cochrane"[All Fields] AND "database"[All Fields] AND "syst"[All Fields] AND "rev"[All Fields]) OR "cochrane database syst rev"[All Fields])) | 3177 |
| 2 | Scopus | "*in vitro*"[All Fields] AND ("systematic review"[All fields] OR "meta-analysis"[All fields] OR "meta-analysis"[Publication Type] OR ("Cochrane Database Syst Rev"[Journal] OR ("Cochrane"[All Fields] AND "database"[All Fields] AND "syst"[All Fields] AND "rev"[All Fields]) OR "cochrane database syst rev"[All Fields])) | 8482 |
| 3 | Web of science  (ISI) | "*in vitro*"[All Fields] AND ("systematic review"[All fields] OR "meta-analysis"[All fields] OR "meta-analysis"[Publication Type] OR ("Cochrane Database Syst Rev"[Journal] OR ("Cochrane"[All Fields] AND "database"[All Fields] AND "syst"[All Fields] AND "rev"[All Fields]) OR "cochrane database syst rev"[All Fields])) | 98 |
| 4 | Virtual Health Library (VHL) | "*in vitro*"[All Fields] AND ("systematic review"[All fields] OR "meta-analysis"[All fields] OR "meta-analysis"[Publication Type] OR ("Cochrane Database Syst Rev"[Journal] OR ("Cochrane"[All Fields] AND "database"[All Fields] AND "syst"[All Fields] AND "rev"[All Fields]) OR "cochrane database syst rev"[All Fields])) | 0 |

Table S2. Detailed search strategy for each database search

The search terms were used in Google search engine:

- "in vitro" AND “methodological quality”

- "in vitro" AND “risk of bias"

- "in vitro" AND “critical appraisal"

- "in vitro" AND “validity"

- "in vitro" AND “tool"

- "in vitro" AND “item"

- "in vitro" AND “bias"

- "in vitro" AND “confounding"

- "in vitro" AND “checklist"

- "in vitro" AND “scale"

Our search included the websites as follows, accessed October 1st 2016.

- Critical Appraisal Skills Programme (CASP; <http://www.casp-uk.net/>),

- Scottish Intercollegiate Guidelines Network (SIGN; <http://www.sign.ac.uk/>)

- National Institute for Clinical Excellence (NICE; <http://www.nice.org.uk/>)

- Cochrane Handbook (http://handbook.cochrane.org/)

- Joanna Briggs Institute (JBI) Reviewers Manual (http://www.joannabriggs.org/sumari.html)

- Centre for Reviews and Dissemination’s guidance for undertaking SRs in health care (http://www.york.ac.uk/inst/crd/index_guidance.htm)
