# Supplementary material for: Quality assessment tools used in systematic reviews of in vitro studies: A systematic review
Source: BMC Med Res Methodol. 2021 May 8;21:101. doi: 10.1186/s12874-021-01295-w (PMC8106836; doi:10.1186/s12874-021-01295-w)
Supplement: Supplementary file 3 — Additional file 3: Table S3. List of excluded studies. [file 12874_2021_1295_MOESM3_ESM.docx]

**Table S3.** List of excluded studies.

| ID | Author's name | Title | Reason of exclusion |
| --- | --- | --- | --- |
| 1 | F. B. Azizi, K. Imani, M. M. Golshah, A. Safari-Faramani, R. | Effect of bleaching with carbamide peroxide on shear bond strength of orthodontic brackets: A meta-analysis of in vitro studies | fulltext not found |
| 2 | M. B. Bakonyi, S. Budai-Szücs, M. KovÁcs, A. Spaits, T. Samu, G. Csanyi, E. | In vitro skin models in the optimization of skin formulations | fulltext not found |
| 3 | M. R. Peralta-Mamani, D. Duarte, M. A. H. Santiago Junior, J. F. Honório, H. M. | Manual vs. rotary instrumentation in endodontic treatment of permanent teeth: A systematic review and meta-analysis | fulltext not found |
| 4 | E. R. d. C. Silva, F. O. Teixeira, L. G. B. Santos, N. G. L. Felipe, F. A. Santana, H. S. R. Shanmugam, S. Quintans Júnior, L. J. de Souza Araújo, A. A. Nunes, P. S. | Pharmacological Effects of Carvacrol in In vitro Studies: A Review | fulltext not found |
| 5 | Hamdan-Nassar, T., Bellot-Arcís, C., Paredes-Gallardo, V., García-Sanz, V., Pascual-Moscardó, A., Almerich-Silla, J. M. and Montiel-Company, J. M. | Effect of 2% Chlorhexidine Following Acid Etching on Microtensile Bond Strength of Resin Restorations: A Meta-Analysis | fulltext not found |
| 6 | Buck, TM; Wijnholds, J | Recombinant Adeno-Associated Viral Vectors (rAAV)-Vector Elements in Ocular Gene Therapy Clinical Trials and Transgene Expression and Bioactivity Assays | not in vitro study |
| 7 | Paolucci, T; Pezzi, L; Centra, AM; Giannandrea, N; Bellomo, RG; Saggini, R | Electromagnetic Field Therapy: A Rehabilitative Perspective in the Management of Musculoskeletal Pain - A Systematic Review | not in vitro study |
| 8 | [Anonymous] | 46th ESAO Congress 3-7 September 2019 Hannover, Germany Abstracts | not in vitro study |
| 9 | Fiorillo, L | Chlorhexidine Gel Use in the Oral District: A Systematic Review | not in vitro study |
| 10 | Akheruzzaman, M; Hegde, V; Dhurandhar, NV | Twenty-five years of research about adipogenic adenoviruses: A systematic review | not in vitro study |
| 11 | Driessen, S; Napp, A; Schmiedchen, K; Kraus, T; Stunder, D | Electromagnetic interference in cardiac electronic implants caused by novel electrical appliances emitting electromagnetic fields in the intermediate frequency range: a systematic review | not in vitro study |
| 12 | Baak, NA; Cantineau, AEP; Farquhar, C; Brison, DR | Temperature of embryo culture for assisted reproduction | not in vitro study |
| 13 | Vijayalaxmi; Prihoda, TJ | Comprehensive Review of Quality of Publications and Meta-analysis of Genetic Damage in Mammalian Cells Exposed to Non-Ionizing Radiofrequency Fields | not in vitro study |
| 14 | Akhtar, MA; Agrawal, R; Brown, J; Sajjad, Y; Craciunas, L | Thyroxine replacement for subfertile women with euthyroid autoimmune thyroid disease or subclinical hypothyroidism | not in vitro study |
| 15 | Seetaloo, AD; Aumeeruddy, MZ; Kannan, RRR; Mahomoodally, MF | Potential of traditionally consumed medicinal herbs, spices, and food plants to inhibit key digestive enzymes geared towards diabetes mellitus management - A systematic review | not in vitro study |
| 16 | Pandya, RK; Shah, M; Shroff, Y; Vyas, M | Effect of Cryopreservation on the Structural and Functional Integrity of Human Periodontal Ligament Stem Cells: A Systematic Review | not in vitro study |
| 17 | Chen, K; Cheng, MP; Hammond, SP; Einsele, H; Marty, FM | Antiviral prophylaxis for cytomegalovirus infection in allogeneic hematopoietic cell transplantation | not in vitro study |
| 18 | Vuka, I; Vucic, K; Repic, T; Hamzic, LF; Sapunar, D; Puljak, L | Electrical Stimulation of Dorsal Root Ganglion in the Context of Pain: A Systematic Review of In Vitro and In Vivo Animal Model Studies | not in vitro study |
| 19 | Yaqoob, A; Al Shehrani, I; Alfarsi, M; Baba, S; Kanji, MA; Hussain, MW | PANORAMA OF IMPRESSION TECHNIQUES IN FIXED PARTIAL DENTURES. A SYSTEMATIC REVIEW | not in vitro study |
| 20 | Dunn, SE; Vicini, JL; Glenn, KC; Fleischer, DM; Greenhawt, MJ | The allergenicity of genetically modified foods from genetically engineered crops A narrative and systematic review | not in vitro study |
| 21 | Iversen, ML; Seyer-Hansen, M; Forman, A | Does surgery for deep infiltrating bowel endometriosis improve fertility? A systematic review | not in vitro study |
| 22 | Hernandez, AF; Tsatsakis, AM | Human exposure to chemical mixtures: Challenges for the integration of toxicology with epidemiology data in risk assessment | not in vitro study |
| 23 | El Hajj, MS; Turgeon, RD; Wilby, KJ | Ceftaroline fosamil for community-acquired pneumonia and skin and skin structure infections: a systematic review | not in vitro study |
| 24 | Miron, RJ; Fujioka-Kobayashi, M; Bishara, M; Zhang, YF; Hernandez, M; Choukroun, J | Platelet-Rich Fibrin and Soft Tissue Wound Healing: A Systematic Review | not in vitro study |
| 25 | Noto, V; Harrity, C; Walsh, D; Marron, K | The impact of FMR1 gene mutations on human reproduction and development: a systematic review | not in vitro study |
| 26 | Bech, NH; Hulst, AH; Spuijbroek, JA; van Leuken, LLA; Haverkamp, D | Perioperative pain management in hip arthroscopy; what options are there? | not in vitro study |
| 27 | Cadavid, D; Auwaerter, PG; Rumbaugh, J; Gelderblom, H | Antibiotics for the neurological complications of Lyme disease | not in vitro study |
| 28 | Ching, C., Orubu, E. S. F., Wirtz, V. J. and Zaman, M. H. | Bacterial antibiotic resistance development and mutagenesis following exposure to subminimal inhibitory concentrations of fluoroquinolones in vitro: a systematic literature review protocol | not in vitro study |
| 29 | Vardakas, K. Z., Athanassaki, F., Pitiriga, V. and Falagas, M. E. | Clinical relevance of in vitro synergistic activity of antibiotics for multidrug-resistant Gram-negative infections: A systematic review | not in vitro study |
| 30 | Pérez-Recalde, M., Ruiz Arias, I. E. and Hermida, É B. | Could essential oils enhance biopolymers performance for wound healing? A systematic review | not in vitro study |
| 31 | Ho, N. C., Ebramzadeh, E. and Sangiorgio, S. N. | Preclinical biomechanical testing models for the tibiotalar joint and its replacements: A systematic review | not in vitro study |
| 32 | Carvalho, P. H. A., Moura, L. B., Trento, G. S., Holzinger, D., Gabrielli, M. A. C., Gabrielli, M. F. R. and Pereira Filho, V. A. | Surgically assisted rapid maxillary expansion: a systematic review of complications | not in vitro study |
| 33 | Khanjani, S., Sedigh Ebrahim-Saraie, H., Malekzadegan, Y., Halaji, M. and Mojtahedi, A. | Systematic review of antibacterial activity of eravacycline: A novel fluorocycline against clinically obtained Gram-negative bacteria | not in vitro study |
| 34 | L. T. T. Qin, R. X. Lin, P. Li, Q. Yang, H. Luo, D. Z. Chen, G. He, Y. Li, P. | Biological function of UCA1 in hepatocellular carcinoma and its clinical significance: Investigation with in vitro and meta-analysis | Not pure in vitro |
| 35 | Verma, UP; Yadav, RK; Dixit, M; Gupta, A | Platelet-rich fibrin: A paradigm in periodontal therapy u A systematic review | Not pure in vitro |
| 36 | Del Fabbro, M; Bortolin, M; Taschieri, S; Ceci, C; Weinstein, RL | Antimicrobial properties of platelet-rich preparations. A systematic review of the current pre-clinical evidence | Not pure in vitro |
| 37 | Carneiro Pereira, A. L., Bezerra de Medeiros, A. K., de Sousa Santos, K., Oliveira de Almeida, É, Seabra Barbosa, G. A. and da Fonte Porto Carreiro, A. | Accuracy of CAD-CAM systems for removable partial denture framework fabrication: A systematic review | Not pure in vitro |
| 38 | Varshney, S., Dwivedi, A. and Pandey, V. | Antimicrobial effects of various platelet rich concentrates-vibes from in-vitro studies-a systematic review | Not pure in vitro |
| 39 | Baptista, A., Gonçalves, R. V., Bressan, J. and do Carmo Gouveia Pelúzio, M. | Antioxidant and antimicrobial activities of crude extracts and fractions of cashew (Anacardium occidentale L.), cajui (Anacardium microcarpum), and pequi (Caryocar brasiliense C.): A systematic review | Not pure in vitro |
| 40 | Kachhara, S., Nallaswamy, D., Ganapathy, D. M., Sivaswamy, V. and Rajaraman, V. | Assessment of intraoral scanning technology for multiple implant impressions - A systematic review and meta-analysis | Not pure in vitro |
| 41 | Bellinaso, M. D., Soares, F. Z. M. and Rocha, R. O. | Do bulk-fill resins decrease the restorative time in posterior teeth? A systematic review and meta-analysis of in vitro studies | Not pure in vitro |
| 42 | Schwarzer, S., James, G. A., Goeres, D., Bjarnsholt, T., Vickery, K., Percival, S. L., Stoodley, P., Schultz, G., Jensen, S. O. and Malone, M. | The efficacy of topical agents used in wounds for managing chronic biofilm infections: A systematic review | Not pure in vitro |
| 43 | Crowe, W., Allsopp, P. J., Watson, G. E., Magee, P. J., Strain, J. J., Armstrong, D. J., Ball, E. and McSorley, E. M. | Mercury as an environmental stimulus in the development of autoimmunity – A systematic review | Not pure in vitro |
| 44 | Sánchez, M., González-Burgos, E., Iglesias, I., Lozano, R. and Gómez-Serranillos, M. P. | The pharmacological activity of camellia sinensis (L.) kuntze on metabolic and endocrine disorders: A systematic review | Not pure in vitro |
| 45 | Keerthana, S. and Kumar, A. | Potential risks and benefits of zinc oxide nanoparticles: a systematic review | Not pure in vitro |
| 46 | Al-Dabbagh, R. A. | Survival and success of endocrowns: A systematic review and meta-analysis | Not pure in vitro |
| 47 | Kojom Foko, L. P., Eya'Ane Meva, F., Eboumbou Moukoko, C. E., Ntoumba, A. A., Ngaha Njila, M. I., Belle Ebanda Kedi, P., Ayong, L. and Lehman, L. G. | A systematic review on anti-malarial drug discovery and antiplasmodial potential of green synthesis mediated metal nanoparticles: Overview, challenges and future perspectives | Not pure in vitro |
| 48 | Szava, DT; Szava, I; Ormenisan, A; Comaneanu, RM; Hancu, V; Szekely, M | Experimental Analysis of Dental Implant Biomechanics Related to Vertical and Horizontal Dimensions of the Fixating Substrate Using Digital Image Correlation Method | Not SR |
| 49 | Katchan, V; David, P; Shoenfeld, Y | Cannabinoids and autoimmune diseases: A systematic review | Not SR |
| 50 | Chen, S., Zhu, Q., Sun, H., Zhang, Y., Tighe, S., Xu, L. and Zhu, Y. | Advances in culture, expansion and mechanistic studies of corneal endothelial cells: A systematic review | Not SR |
| 51 | Sauter, E. R. | Cancer prevention and treatment using combination therapy with natural compounds | Not SR |
| 52 | Grandhi, R. K., Lee, S. and Abd-Elsayed, A. | Does Opioid Use Cause Angiogenesis and Metastasis? | Not SR |
| 53 | Svante Twetman, Susanna Axelsson, Gunnar Dahlén, Ivar Espelid, Ingegerd Mejàre, Anders Norlund, Sofia Tranæus | Adjunct methods for caries detection: A systematic review of literature | not in vitro study |
| 54 | Leonard A. Mermel and Neha Alang | Adverse effects associated with ethanol catheter lock solutions: a systematic review | Not pure in vitro |
| 55 | Zohaib Akram, Saud A. Aziz Al-Shareef, Umer Daood, Faris Yahya Asiri, Altaf H. Shah, M. Ayedh AlQahtani, Fahim Vohra, MClinDent and Fawad Javed | Bactericidal Efficacy of Photodynamic Therapy Against Periodontal Pathogens in Periodontal Disease: A Systematic Review | Not pure in vitro |
| 56 | Wolf Petersen, Andree Ellermann, Thore Zantop, Ingo Volker Rembitzki, Hartmut Semsch, Christian Liebau, Raymond Best | Biomechanical effect of unloader braces for medial osteoarthritis of the knee: a systematic review (CRD 42015026136) | not in vitro study |
| 57 | Hana Polanska, Martina Raudenska, Jaromir Gumulec, Marketa Sztalmachova, Vojtech Adam, Rene Kizek, Michal Masarik | Clinical significance of head and neck squamous cell cancer biomarkers | Not SR |
| 58 | Jianjun li, lu liang, Yongru liu, Yihuan luo, Xiaona liang, Dianzhong luo, Zhenbo Feng, Yiwu Dang, lihua Yang, Gang chen | Clinicopathological significance of STAT4 in hepatocellular carcinoma and its effect on cell growth and apoptosis | Not SR |
| 59 | Mical Paul, Leonard Leibovici | Combination Antimicrobial Treatment Versus Monotherapy: The Contribution of Meta - analyses | Not SR |
| 60 | Gianluca Occhi, Susi Barollo, Daniela Regazzo, Loris Bertazza, Francesca Galuppini, Vincenza Guzzardo, Marie Lise Jaffrain-Rea, Federica Vianello,Denis Ciato, Filippo Ceccato, Sara Watutantrige-Fernando, Andrea Bisognin, Stefania Bortoluzzi, Gianmaria Pennelli, Marco Boscaro, Carla Scaroni, Caterina Mian | A constitutive active MAPK/ERK pathway due to BRAFV600E positively regulates AHR pathway in PTC | Not SR |
| 61 | Esam Omar |    \| Current concepts and future of noninvasive procedures for diagnosing oral squamous cell carcinoma - a systematic review \| \| --- \| | not in vitro study |
| 62 | Michelle A. Scott, Virginia T. Nguyen, Benjamin Levi and Aaron W. James | Current methods of adipogenic differentiation of mesenchymal stem cells | Not SR |
| 63 | Victor Eduardo de Souza Batista, Joel Ferreira Santiago Junior, Daniel Augusto de Faria Almeida, Leonardo Ferreira de Toledo Piza Lopes, Fellippo Ramos Verri & Eduardo Piza Pellizzer | The effect of offset implant configuration on bone stress distribution a systematic review | Not pure in vitro |
| 64 | Anna Louropoulou, Dagmar E. Slot, Fridus Van der Weijden | The effects of mechanical instruments on contaminated titanium dental implant surfaces: a systematic review | not in vitro study |
| 65 | Abeer El-sayed Elembaby | The effects of mouth rinses on the color stability of resin based restorative materials | Not SR |
| 66 | Monica Suet Ying Ng, Angela Suet Yeung Ng, Jessica Chan, John-Paul Tung, John Francis Frase | Effects of packed red blood cell storage duration on post transfusion clinical outcomes a meta-analysis and systematic review | Not pure in vitro |
| 67 | Henk van der Worp, Inge van den Akker-Scheek, Hans van Schie, Johannes Zwerver | ESWT for tendinopathy technology and clinical implications | Not SR |
| 68 | John Fredy Cuervo-Perez, Julián Camilo Arango, Jaiberth Antonio Cardona-Arias | Evaluation of techniques in vitro immune to the diagnosis of allergy meta-analysis 2000-2012 | Not pure in vitro |
| 69 | Marian Jedrych, Katarzyna Borowska, Ryszard Galus, Barbara Jodłowska-Jedrych | The evaluation of the biomedical effectiveness of polyamidoamine dendrimers generation 4.0 as a drug and as drug carriers a systematic review and meta-analysis | not in vitro study |
| 70 | Yau-Hua Yu, Hsu-Ko Kuo, Kuo-Wei Chang | The Evolving Transcriptome of Head and Neck Squamous Cell Carcinoma: A Systematic Review | not in vitro study |
| 71 | Elizabeth A. Guancial, Joaquim Bellmunt, Shuyuan Yeh, Jonathan Rosenberg and David M. Berman | The Evolving Understanding of MicroRNA in Bladder Cancer | Not pure in vitro |
| 72 | Guiping Wang, Yun Ye, Xiaoqin Yang, Hongying Liao, Canguo Zhao, Shuang Liang | Expression Based In Silico Screening of Candidate Therapeutic Compounds for Lung Adenocarcinoma | Not SR |
| 73 | MARC GHANNOUM, CHRISTOPHER YATES, TAIS F. GALVAO, KEVIN M. SOWINSKI, THI HAI VÂN VO, ANDREW COOGAN, SOPHIE GOSSELIN, VALERY LAVERGNE, THOMAS D. NOLIN, and ROBERT S. HOFFMAN, ON BEHALF OF THE EXTRIP WORKGROUP | Extracorporeal treatment for carbamazepine poisoning: Systematic review and recommendations from the EXTRIP workgroup | Not pure in vitro |
| 74 | P Pranau Vanajasan, Malarvizhi Dhakshinamoorthy, CV Subba Rao |    \| Factors affecting the bond strength of self-etch adhesives: A meta-analysis of literature \| \| --- \| | not in vitro study |
| 75 | Isabelle Cleynen, Peter Juni, Geertruida E. Bekkering, Eveline Nuesch, Camila T. Mendes, Stefanie Schmied, Stefan Wyder, Eliane Kellen, Peter M. Villiger, Paul Rutgeerts, Severine Vermeire, Daniel Lottaz | Genetic evidence supporting the association of protease and protease inhibitor genes with inflammatory bowel disease a systematic review | not in vitro study |
| 76 | Roeland Van Wijk | The in vitro evidence for an effect of high homeopathic potencies-a systematic review of the literature | Not SR |
| 77 | Jamie I. MacPherson, Ben Sidders, Stefan Wieland, Jin Zhong, Paul Targett-Adams, Volker Lohmann, Perdita Backes, Oona Delpuech-Adams, Francis Chisari, Marilyn Lewis, Tanya Parkinson, David L. Robertson | An Integrated Transcriptomic and Meta-Analysis of Hepatoma Cells Reveals Factors That Influence Susceptibility to HCV Infection | Not SR |
| 78 | Ludmila Madeira Cardoso Pavan, Daniela Fortunato Rêgo, Silvia Taveira Elias, Graziela De Luca Canto, Eliete Neves Silva Guerra | In vitro Anti-Tumor Effects of Statins on Head and Neck Squamous Cell Carcinoma A Systematic Review | Not pure in vitro |
| 79 | Nicholas Mowbray, James Ansell, Neil Warren, Pete Wall, Jared Torkington | Is surgical smoke harmful to theater staff? A systematic review | not in vitro study |
| 80 | HUGO SOUSA, ALEXANDRA M. SANTOS, DANIELA PINTO and RUI MEDEIROS | Is the p53 codon 72 polymorphism-a key biomarker for cervica | not in vitro study |
| 81 | Abraham T. Girgih, Ifeanyi D. Nwachukwu, Fida Hasan, Tayo N. Fagbemi, Tom Gill and Rotimi E. Aluko | Kinetics of the inhibition of renin and angiotensin I-converting enzyme by cod (Gadus morhua) protein hydrolysates and their antihypertensive effects in spontaneously hypertensive rats | Not SR |
| 82 | R. Kumar, M. Griffin, G. Adigbli, N. Kalavrezos and P. E. M. Butler | Lipotransfer for radiation-induced skin fibrosis | not in vitro study |
| 83 | Noemı Carranza, Victoria Ramos, Francisca G. Lizana, Jorge Garcıa, Alejando del Pozo and Jose Luis Monteagudo | A literature review of transmission effectiveness and electromagnetic compatibility in home telemedicine environments to evaluate safety and security | not in vitro study |
| 84 | Gabriel A. March, Miguel A. Bratos | A meta-analysis of in vitro antibiotic synergy against Acinetobacter baumannii | Not pure in vitro |
| 85 | Slavomira Doktorovova, Eliana B. Souto, Amélia M. Silva | Nanotoxicology applied to solid lipid nanoparticles and nanostructured lipid carriers: a systematic review of in vitro data | Not pure in vitro |
| 86 | Nguyen Phuoc Long, Wun Jun Lee, nguyen truong Huy, seul Ji Lee, Jeong Hill Park and sung Won Kwon | Novel Biomarker Candidates for Colorectal Cancer Metastasis A Meta-analysis of In Vitro Studies | Not SR |
| 87 | Marília Afonso Rabelo BUZALAF, Angélica Reis HANNAS, Ana Carolina MAGALHÃES, Daniela RIOS, Heitor Marques HONÓRIO, Alberto Carlos Botazzo DELBEM | pH cycling models for in vitro evaluation of the efficacy of fluoridated dentifrices for caries control strengths and limitations | Not pure in vitro |
| 88 | Arno C. Gutleb | Potential of In Vitro Methods for Mechanistic Studies of Particulate Matter Induced Cardiopulmonary Toxicity | Not SR |
| 89 | Penelope AE Main, Manya T Angley, Catherine E O’Doherty, Philip Thomas and Michael Fenech | The potential role of the antioxidant and detoxification properties of glutathione in autism spectrum disorders a systematic review and meta-analysis | Not pure in vitro |
| 90 | Ilias I. Siempos, Konstantinos Z. Vardakas, Christos E. Kyriakopoulos, Theodora K. Ntaidou and Matthew E. Falagas | Predictors of mortality in adult patients with ventilator associated pneumonia a meta-analysis | Not pure in vitro |
| 91 | Lygia T Budnik, Stefan Kloth, Marcial Velasco-Garrido and Xaver Baur | Prostate cancer and toxicity from critical use exemptions of methyl bromide environmental protection helps protect against human health risks | Not pure in vitro |
| 92 | A. Vlassopoulos, M. E. J. Lean and E. Combet | Protein phenolic interactions and inhibition of glycation combining a systematic review and experimental models for enhanced physiological relevance | not in vitro study |
| 93 | Yusuf Assem, Ralph J. Mobbs, Matthew H. Pelletier, Kevin Phan, William R. Walsh | Radiological and clinical outcomes of novel TiPEEK combined spinal fusion cages a systematic review and preclinical evaluation | not in vitro study |
| 94 | Lucietta Betti, Grazia Trebbi, Michela Zurla, Daniele Nani, Maurizio Peruzzi, and Maurizio Brizzi | A review of three simple plant models and corresponding statistical tools for basic | Not SR |
| 95 | Sunil Apsingi, Anthony M. J. Bull, David J. Deehan, Andrew A. Amis | Review femoral tunnel placement for PCL reconstruction in relation to the PCL fibre bundle attachments | not in vitro study |
| 96 | Jafar Kolahi, Ahmad Soolari | Rinsing with chlorhexidine gluconate solution after brushing and flossing teeth a systematic review of effectiveness | not in vitro study |
| 97 | Gui Han Lee, Alan Askari, George Malietzis, David Bernardo, Susan K. Clark, Stella C. Knight, and Hafid Omar Al-Hassi | The role of CD40 expression in dendritic cells in cancer biology a systematic review | Not pure in vitro |
| 98 | Matthew E Falagas, Drosos E, Karageorgopoulos, Georgia G, Georgantzi, Chunguang Sun, Rui Wang and Petros I Rafailidis | Susceptibility of Gramnegative bacteria to isepamicin a systematic review | Not pure in vitro |
| 99 | Hanna Nebenzahl-Guimaraes, Karen R. Jacobson, Maha R. Farhat and Megan B. Murray | Systematic review of allelic exchange experiments aimed at identifying mutations that confer drug resistance in Mycobacterium tuberculosis | Not pure in vitro |
