# Supplementary material for: Quality assessment tools used in systematic reviews of in vitro studies: A systematic review
Source: BMC Med Res Methodol. 2021 May 8;21:101. doi: 10.1186/s12874-021-01295-w (PMC8106836; doi:10.1186/s12874-021-01295-w)
Supplement: Supplementary file 4 — Additional file 4: Table S4. List of included articles using QA tools in in vitro SRs/MAs. [file 12874_2021_1295_MOESM4_ESM.docx]

Table S4. List of included articles using QA tools in *in vitro* SRs.

| Author/publication year | Country of the first author | Study topic | Included studies (*n*) | Quality tool used | Conducting meta-analysis | QA | QA tool used |
| --- | --- | --- | --- | --- | --- | --- | --- |
| Strauss/2019 (1) | Austria | Bioactivity | 37 | PRISMA | N | N | NR |
| Magrin/2020 (2) | Austria | Bioactivity | 10 | PRISMA | N | Y | World Cancer Research Fund/  University of Bristol for cell line |
| Pavan/2015 (3) | Brazil | Bioactivity | 14 | PRISMA | N | Y | GRADE |
| Diefenbach/2017 (4) | Brazil | Bioactivity | 11 | PRISMA | N | Y | Joanna Briggs Institute  Clinical Appraisal Checklist |
| Rêgo/2017 (5) | Brazil | Bioactivity | 11 | PRISMA | N | Y | GRADE |
| Oliveira/2018 (6) | Brazil | Bioactivity | 22 | PRISMA | N | N | NR |
| Hellwig/2019 (7) | Brazil | Bioactivity | 35 | N | N | N | NR |
| Abreu/2019 (8) | Brazil | Bioactivity | 55 | NR | N | Y | OHAT |
| Silva/2019 (9) | Brazil | Bioactivity | 19 | N | N | N | NR |
| Picco/2019 (10) | Brazil | Bioactivity | 11 | PRISMA | N | N | NR |
| Sanches/2020 (11) | Brazil | Bioactivity | 7 | PRISMA | N | Y | ToxRTool |
| Xiao/2011 (12) | China | Bioactivity | 19 | N | N | Y | Referencing CRH and  EBM Evidence Pyramid |
| Ni/2015 (13) | China | Bioactivity | 101 | N | Y | N | NR |
| Asweto/2017 (14) | China | Bioactivity | 14 | OHAT | N | Y | Following previous description  of Samuel *et al.* |
| Li/2017 (15) | China | Bioactivity | 37 | N | N | N | NR |
| Munitić/2019 (16) | Croatia | Bioactivity | 37 | PRISMA | N | Y | NR |
| Saravanan/2020 (17) | Ethiopia | Bioactivity | 53 | PRISMA | N | N | NR |
| Khanafer/2017 (18) | France | Bioactivity | 182 | N | N | N | NR |
| Pummer/2017 (19) | Germany | Bioactivity | 34 | PRISMA | N | N | NR |
| K/2017 (20) | India | Bioactivity | 17 | STROBE | N | N | NR |
| Moradi/2016 (21) | Iran | Bioactivity | 42 | N | N | N | NR |
| Samiei/2016 (22) | Iran | Bioactivity | 15 | PRISMA | Y | N | NR |
| Hasannejad-Bibalan/2019 (23) | Iran | Bioactivity | 18 | N | Y | Y | STROBE |
| Mansourian/2020 (24) | Iran | Bioactivity | 19 | N | Y | N | NR |
| Khaledi/2020 (25) | Iran | Bioactivity | 17 | N | N | Y | Developed by authors |
| Zusman/2013 (26) | Israel | Bioactivity | 54 | N | Y | N | NR |
| Gizzo/2015 (27) | Italy | Bioactivity | 20 | N | N | N | NR |
| Gianfredi/2017 (28) | Italy | Bioactivity | 14 | N | N | N | NR |
| Gianfredi/2017 (29) | Italy | Bioactivity | 16 | PRISMA | Y | N | NR |
| Zhurakivska/2018 (30) | Italy | Bioactivity | 16 | PRISMA | N | N | NR |
| Lemma/2017 (31) | Japan | Bioactivity | 58 | PRISMA | N | N | NR |
| Puidokas/2019 (32) | Lithuania | Bioactivity | 14 | PRISMA | N | Y | OHAT |
| Almoudi/2018 (33) | Malaysia | Bioactivity | 17 | PRISMA | N | N | NR |
| Lim/2020 (34) | Malaysia | Bioactivity | 14 | PRISMA | N | Y | Cochrane Risk of Bias tool |
| Hlashwayo/2020 (35) | Mozambique | Bioactivity | 14 | PRISMA | N | N | NR |
| Montano/2010 (36) | Netherlands | Bioactivity | 79 | N | Y | N | NR |
| Golbach/2016 (37) | Netherlands | Bioactivity | 33 | N | Y | Y | Developed by authors |
| Dobbenga/2016 (38) | Netherlands | Bioactivity | 20 | N | N | N | NR |
| Hoppenbrouwers/2017 (39) | Netherlands | Bioactivity | 25 | PRISMA | N | N | NR |
| Pardo-Aldave/2019 (40) | Peru | Bioactivity | 11 | PRISMA | N | Y | Joanna Briggs Institute  Clinical Appraisal Checklist |
| Alshwaimi/2016 (41) | Saudi Arabia | Bioactivity | 24 | PRISMA | N | Y | Following previous description of  Onofre *et al.* and Montagner *et al.* |
| Alasqah/2019 (42) | Saudi Arabia | Bioactivity | 7 | PRISMA | N | N | NR |
| Al-Hamdan/2019 (43) | Saudi Arabia | Bioactivity | 5 | PRISMA | N | N | NR |
| Napotnik/2016 (44) | Slovenia | Bioactivity | 203 | N | N | Y | Cochrane Risk of Bias tool |
| Kaufmann/2018 (45) | Switzerland | Bioactivity | 3 | PRISMA | N | N | NR |
| Kumar/2016 (46) | UK | Bioactivity | 70 | PRISMA | N | N | NR |
| Jabran/2018 (47) | UK | Bioactivity | 62 | N | N | N | NR |
| Holliday/2019 (48) | UK | Bioactivity | 42 | PRISMA | N | Y | CONSORT |
| Scott/2019 (49) | UK | Bioactivity | 8 | PRISMA | N | N | NR |
| Prpa/2020 (50) | UK | Bioactivity | 120 | PRISMA | N | Y | Cochrane Risk of Bias tool |
| Ting/2016 (51) | USA | Bioactivity | 26 | N | N | N | NR |
| Chemaly/2019 (52) | USA | Bioactivity | 210 | N | N | N | NR |
| Tam/2020 (53) | Vietnam | Bioactivity | 10 | PRISMA | N | Y | ToxRTool |
| Liu/2018 (54) | Austrialia | Biology | 88 | N | Y | Y | Nature Publication Quality  Improvement Project (NPQIP) study |
| Lemos/2017 (55) | Brazil | Biology | 37 | N | N | N | NR |
| Pacheco/2019 (56) | Brazil | Biology | 5 | N | N | Y | Cochrane Risk of Bias tool |
| Chierrito/2019 (57) | Brazil | Biology | 16 | PRISMA | N | Y | SYCLE |
| Silveira/2020 (58) | Brazil | Biology | 10 | N | N | Y | Developed by authors |
| Rahman/2016 (59) | Brunei | Biology | 155 | N | N | Y | Modifying Quality Assessment Tool  for Studies with Diverse Designs (QATSDD) |
| Ren/2016 (60) | China | Biology | 21 | N | N | N | NR |
| Li/2019 (61) | China | Biology | 26 | PRISMA | N | Y | ARRIVE |
| Kirkegaard/2017 (62) | Denmark | Biology | 5 | PRISMA | N | Y | NOS |
| Bleuel/2015 (63) | Germany | Biology | 33 | PRISMA | N | N | NR |
| Dissemond/2020 (64) | Germany | Biology | 107 | N | N | N | NR |
| Jayanegara/2018 (65) | Indonesia | Biology | 16 | N | N | N | NR |
| Tabatabaei-Malazy/2012 (66) | Iran | Biology | 49 | N | N | N | NR |
| Mohammadrezaei/2018 (67) | Iran | Biology | 82 | PRISMA | N | N | NR |
| Franzoni/2017 (68) | Ireland | Biology | 17 | N | N | N | NR |
| Rotelli/2015 (69) | Italy | Biology | 46 | N | N | N | NR |
| Maccarana/2016 (70) | Italy | Biology | 47 | N | N | N | NR |
| Veronesi/2019 (71) | Italy | Biology | 20 | PRISMA | N | N | NR |
| Gentile/2020 (72) | Italy | Biology | 341 | PRISMA | N | Y | NOS |
| Contreras-Ochoa/2012 (73) | Mexico | Biology | 15 | N | N | N | NR |
| Peplow/2013 (74) | New Zealand | Biology | 24 | N | N | N | NR |
| Tong/2015 (75) | New Zealand | Biology | 47 | N | N | N | NR |
| Vasyutin/2019 (76) | Russia | Biology | 58 | PRISMA | N | N | NR |
| Garcia-Sanz/2018 (77) | Spain | Biology | 52 | PRISMA | Y | Y | Following previous description of Onofre et al. |
| Bryant/2018 (78) | UK | Biology | 14 | N | N | Y | Standard Quality Assessment Criteria  for Evaluating Primary Research Papers  from a Variety of Fields |
| Chew/2020 (79) | UK | Biology | 603 | N | N | N | NR |
| Bahsoun/2019 (80) | UK | Biology | 41 | N | N | N | NR |
| Daltona/2019 (81) | UK | Biology | 12 | PRISMA | N | N | NR |
| Arilla/2015 (82) | USA | Biology | 48 | N | N | Y | Developed by authors |
| Bates/2015 (83) | USA | Biology | 77 | N | Y | N | NR |
| Slette/2016 (84) | USA | Biology | 10 | PRISMA | N | N | NR |
| Nawafleh/2016 (85) | Australia | Dentistry | 19 | N | N | N | NR |
| Shahmiri/2017 (86) | Australia | Dentistry | 57 | PRISMA | N | N | NR |
| Elshiyab/2017 (87) | Australia | Dentistry | 25 | PRISMA | N | N | NR |
| Thammajaruk/2018 (88) | Australia | Dentistry | 161 | N | N | N | NR |
| Kulkarni/2020 (89) | Australia | Dentistry | 9 | PRISMA | N | Y | Cochrane Risk of Bias tool |
| Schmid-Schwap/2011 (90) | Austria | Dentistry | 16 | N | Y | N | NR |
| Wehner/2020 (91) | Austria | Dentistry | 11 | PRISMA | Y | Y | Developed by authors |
| Swimberghe/2018 (92) | Belgium | Dentistry | 77 | PRISMA | N | N | NR |
| Osmanovic/2018 (93) | Bosnia and Herzegovina | Dentistry | 15 | PRISMA | N | Y | OHAT |
| Chaves/2012 (94) | Brazil | Dentistry | 20 | N | Y | N | NR |
| Costa/2013 (95) | Brazil | Dentistry | 8 | N | Y | N | NR |
| Sarkis-Onofre/2014 (96) | Brazil | Dentistry | 22 | N | Y | Y | Developed by authors |
| Bernades/2014 (97) | Brazil | Dentistry | 20 | N | N | N | NR |
| Kaizer/2014 (98) | Brazil | Dentistry | 28 | PRISMA | N | N | NR |
| Moraes/2015 (99) | Brazil | Dentistry | 23 | PRISMA | Y | Y | Following previous description  of Onofre *et al.* |
| Rosa/2015 (100) | Brazil | Dentistry | 10 | PRISMA | Y | Y | Following previous description  of Onofre *et al.* |
| Pereiraa/2015 (101) | Brazil | Dentistry | 19 | PRISMA | Y | Y | Following previous description  of Onofre *et al.* and Montagner *et al.* |
| Skupien/2015 (102) | Brazil | Dentistry | 34 | PRISMA | Y | N | NR |
| Altmann/2016 (103) | Brazil | Dentistry | 12 | PRISMA and AMSTAR | Y | Y | Developed by authors |
| Soares/2016 (104) | Brazil | Dentistry | 11 | PRISMA | Y | Y | Following previous description  of Onofre *et al.* |
| Aurelio/2016 (105) | Brazil | Dentistry | 44 | PRISMA | Y | Y | Following previous description  of Onofre *et al.* |
| Lenzi/2016 (106) | Brazil | Dentistry | 36 | PRISMA | Y | Y | Following previous description  of Onofre *et al.* |
| Rosa/2016 (107) | Brazil | Dentistry | 39 | PRISMA | N | Y | QUADAS-2 |
| Reis/2017 (108) | Brazil | Dentistry | 10 | N COCHRANE | N | Y | Developed by authors |
| Ferrúa/2017 (109) | Brazil | Dentistry | 222 | PRISMA | N | Y | Developed by authors |
| Silva/2017 (110) | Brazil | Dentistry | 6 | PRISMA | N | Y | Following previous description  of Onofre *et al.* |
| Maske/2017 (111) | Brazil | Dentistry | 85 | PRISMA | N | Y | Following previous description  of Onofre *et al.* |
| Pardal-Peláez/2017 (112) | Brazil | Dentistry | 10 | N | N | N | NR |
| Mello/2017 (113) | Brazil | Dentistry | 15 | PRISMA | Y | N | NR |
| Correa/2018 (114) | Brazil | Dentistry | 6 | PRISMA | Y | Y | Following previous description of Onofre et al. |
| Mello/2018 (115) | Brazil | Dentistry | 23 | PRISMA | N | Y | CASP and MINORS |
| Perroni/2018 (116) | Brazil | Dentistry | 21 | PRISMA | N | Y | CONSORT |
| Carvalho/2018 (117) | Brazil | Dentistry | 18 | PRISMA | Y | Y | CONSORT |
| Caldas/2018 (118) | Brazil | Dentistry | 10 | PRISMA | N | Y | ToxRTool |
| Menezes-Silva/2018 (119) | Brazil | Dentistry | 1 | N | N | Y | CRIS guidelines |
| Marchionatti/2018 (120) | Brazil | Dentistry | 32 | PRISMA | Y | Y | Developed by authors |
| Bohrer/2018 (121) | Brazil | Dentistry | 97 | PRISMA | Y | Y | Following previous description  of Onofre *et al.* |
| Schestatsky/2018 (122) | Brazil | Dentistry | 3 | PRISMA | Y | Y | Following previous description  of Onofre *et al.* |
| Almeida/2018 (123) | Brazil | Dentistry | 32 | PRISMA | Y | Y | Following previous description  of Onofre *et al.* |
| Martins/2018 (124) | Brazil | Dentistry | 7 | PRISMA | Y | Y | Following previous description  of Onofre *et al.* |
| Martins/2018 (125) | Brazil | Dentistry | 11 | PRISMA | Y | Y | Developed by authors |
| Sousa/2018 (126) | Brazil | Dentistry | 21 | PRISMA | N | Y | Following previous description  of Onofre *et al.* |
| Münchow/2018 (127) | Brazil | Dentistry | 36 | PRISMA | Y | Y | Following previous description  of Onofre et al. |
| Pires/2018 (128) | Brazil | Dentistry | 37 | PRISMA | Y | Y | Following previous description  of Onofre *et al.* |
| Fumes/2018 (129) | Brazil | Dentistry | 4 | PRISMA | N | N | NR |
| Cury/2019 (130) | Brazil | Dentistry | 7 | PRISMA | N | Y | Downs and Black |
| Tavares/2019 (131) | Brazil | Dentistry | 21 | PRISMA | N | Y | Following Joanna Briggs Institute Clinical  Appraisal Checklist for Experimental Studies |
| Resende/2019 (132) | Brazil | Dentistry | 35 | PRISMA | N | Y | OHAT |
| Nogueira/2020 (133) | Brazil | Dentistry | 15 | PRISMA | N | Y | Following previous description  of Onofre *et al.* |
| Miranda/2020 (134) | Brazil | Dentistry | 18 | PRISMA | N | Y | Following previous description  of Onofre *et al.* |
| Fonseca/2020 (135) | Brazil | Dentistry | 8 | PRISMA | N | Y | GRADE |
| Leão/2020 (136) | Brazil | Dentistry | 18 | PRISMA | N | Y | MINORS |
| Brandeburski/2020 (137) | Brazil | Dentistry | 20 | PRISMA | N | N | NR |
| Kreve/2020 (138) | Brazil | Dentistry | 13 | PRISMA | N | N | NR |
| Ehsani/2009 (139) | Canada | Dentistry | 19 | N | N | Y | Developed by authors |
| Archambault/2010 (140) | Canada | Dentistry | 11 | QUOROM | N | N | NR |
| Nassar/2011 (141) | Canada | Dentistry | 18 | N | N | N | NR |
| Passos/2014 (142) | Canada | Dentistry | 7 | PRISMA | N | Y | Developed by authors |
| Tan/2018 (143) | China | Dentistry | 14 | PRISMA | Y | Y | Developed by authors |
| Yu/2019 (144) | China | Dentistry | 25 | PRISMA | Y | Y | Following previous description  of Onofre *et al.* |
| Yu/2019 (145) | China | Dentistry | 16 | PRISMA | Y | Y | Following previous description  of Onofre *et al.* |
| Wang/2020 (146) | China | Dentistry | 14 | PRISMA | N | Y | Following previous description  of Onofre *et al.* |
| Razdan/2018 (147) | Denmark | Dentistry | 63 | PRISMA and AMSTAR | N | Y | CONSORT |
| Elkaffas/2019 (148) | Egypt | Dentistry | 13 | N | Y | Y | Developed by authors |
| Janjic/2018 (149) | Germany | Dentistry | 71 | PRISMA | N | N | NR |
| Herbst/2019 (150) | Germany | Dentistry | 85 | PRISMA | Y | N | NR |
| Mustafa/2020 (151) | Germany | Dentistry | 33 | N | N | N | NR |
| Bethke/2020 (152) | Germany | Dentistry | 17 | PRISMA | Y | N | NR |
| Tzanakakis/2016 (153) | Greece | Dentistry | 134 | N | N | N | NR |
| Iliadi/2019 (154) | Greece | Dentistry | 13 | PRISMA | Y | Y | Cochrane Risk of Bias tool |
| Gizani/2020 (155) | Greece | Dentistry | 9 | The Cochrane Handbook for Systematic Reviews of Interventions | Y | Y | Cochrane Risk of Bias tool |
| Solanki/2018 (156) | India | Dentistry | 12 | PRISMA | N | N | NR |
| Ajay/2019 (157) | India | Dentistry | 28 | PRISMA | N | Y | Developed by authors |
| Bangera/2020 (158) | India | Dentistry | 6 | PRISMA | Y | Y | CONSORT |
| Parikh/2020 (159) | India | Dentistry | 13 | PRISMA | N | Y | Developed by authors |
| Pandey/2020 (160) | India | Dentistry | 6 | PRISMA | N | Y | Following previous description  of Sackett *et al.* |
| Jayanegara/2014 (161) | Indonesia | Dentistry | 23 | N | Y | N | NR |
| Shahravan/2007 (162) | Iran | Dentistry | 26 | N | N | N | NR |
| Motamedian/2015 (163) | Iran | Dentistry | 38 | N | N | N | NR |
| Hindy/2017 (164) | Iran | Dentistry | 28 | N | N | Y | Developed by authors |
| Davoudi/2018 (165) | Iran | Dentistry | 11 | PRISMA | N | Y | MINORS |
| Imani/2019 (166) | Iran | Dentistry | 19 | N | N | Y | Cochrane Risk of Bias tool |
| Samiei/2019 (167) | Iran | Dentistry | 20 | NR | N | Y | Developed by authors |
| Davoudi/2019 (168) | Iran | Dentistry | 6 | PRISMA | Y | Y | MINORS |
| Pourhajibagher/2020 (169) | Iran | Dentistry | 13 | PRISMA | Y | Y | Developed by authors |
| Gorman/2016 (170) | Ireland | Dentistry | 26 | PRISMA | N | Y | Following previous description  of Onofre *et al.* |
| Tallarico/2018 (171) | Italy | Dentistry | 16 | PRISMA | N | Y | CONSORT |
| Savoldia/2018 (172) | Italy | Dentistry | 101 | N | N | N | NR |
| Lombardo/2019 (173) | Italy | Dentistry | 36 | N | N | N | NR |
| Corvino/2020 (174) | Italy | Dentistry | 21 | PRISMA | N | Y | SciRAP method |
| Masarwa/2016 (175) | Jordan | Dentistry | 9 | N | Y | Y | Timmer’s Analysis Tool |
| Heintze/2008 (176) | Liechtenstein | Dentistry | 20 | N | N | N | NR |
| Dumbryte/2018 (177) | Lithuania | Dentistry | 7 | PRISMA | Y | Y | Cochrane Risk of Bias tool |
| Western/2017 (178) | Malaysia | Dentistry | 12 | PRISMA | Y | N | NR |
| Nagendrababu/2018 (179) | Malaysia | Dentistry | 19 | PRISMA | N | Y | Joanna Briggs Institute  Clinical Appraisal Checklist |
| Chia/2020 (180) | Malaysia | Dentistry | 13 | N | N | Y | Cochrane Risk of Bias tool |
| Cuevas-Suárez/2018 (181) | Mexico | Dentistry | 59 | PRISMA | Y | Y | Following previous description  of Onofre *et al.* |
| Cuevas-Suárez/2020 (182) | Mexico | Dentistry | 45 | PRISMA | Y | Y | Following previous description  of Onofre *et al.* |
| Behring/2008 (183) | Netherlands | Dentistry | NR | N | N | N | NR |
| Heumen/2008 (184) | Netherlands | Dentistry | 8 | N | N | N | NR |
| Finnema/2010 (185) | Netherlands | Dentistry | 24 | N | Y | N | NR |
| Louropoulou/2015 (186) | Netherlands | Dentistry | 11 | PRISMA | N | Y | Developed by authors |
| Papageorgiou-Kyrana/2020 (187) | Netherlands | Dentistry | 16 | PRISMA | N | N | NR |
| Nilsen/2016 (188) | Norway | Dentistry | 29 | N | N | N | NR |
| Mozynska/2017 (189) | Poland | Dentistry | 23 | PRISMA | N | Y | Developed by authors |
| Kaczor/2018 (190) | Poland | Dentistry | 13 | PRISMA | Y | Y | Following previous description  of Onofre *et al.* |
| Gerula-Szymańska/2020 (191) | Poland | Dentistry | 10 | PRISMA | Y | Y | Following previous description  of Onofre *et al.* |
| Moreira/2015 (192) | Portugal | Dentistry | 31 | N | N | N | NR |
| Pinho/2017 (193) | Portugal | Dentistry | 10 | PRISMA | N | Y | NR |
| Al-Aali/2018 (194) | Saudi Arabia | Dentistry | 8 | PRISMA | Y | N | NR |
| AlFawaz/2019 (195) | Saudi Arabia | Dentistry | 6 | PRISMA | N | Y | Developed by authors |
| Alamri/2020 (196) | Saudi Arabia | Dentistry | 7 | PRISMA | N | Y | Following previous description  of Onofre *et al.* |
| Astudillo-Rubio/2018 (197) | Spain | Dentistry | 24 | PRISMA | Y | Y | Following previous description  of Onofre *et al.* |
| Amesti-Garaizabal/2019 (198) | Spain | Dentistry | 29 | PRISMA | Y | N | NR |
| Sanz/2020 (199) | Spain | Dentistry | 10 | PRISMA | N | Y | CONSORT |
| Papia/2014 (200) | Sweden | Dentistry | 127 | N | N | N | NR |
| Ozcan/2016 (201) | Switzerland | Dentistry | 14 | N | N | N | NR |
| Coray/2016 (202) | Switzerland | Dentistry | 7 | N | Y | N | NR |
| Stefan/2017 (203) | Switzerland | Dentistry | 13 | PRISMA | Y | Y | Cochrane Risk of Bias tool |
| Ozcan/2018 (204) | Switzerland | Dentistry | 57 | PRISMA | N | Y | Developed by authors |
| Yaylali/2015 (205) | Turkey | Dentistry | 9 | PRISMA | Y | Y | Joanna Briggs Institute Clinical  Appraisal Checklist |
| Uzunoglu-Özyürek/2018 (206) | Turkey | Dentistry | 20 | PRISMA | N | Y | Following previous description  of Onofre *et al.* |
| Taha/2017 (207) | UK | Dentistry | 11 | PRISMA | N | Y | Following previous description  of Onofre *et al.* |
| Lee/2008 (208) | USA | Dentistry | 41 | N | N | N | NR |
| Kwon/2020 (209) | USA | Dentistry | 11 | N | N | Y | Cochrane Risk of Bias tool |
| Deng/2016 (210) | China | Diagnosis | 9 | N | Y | Y | QUADAS-2 |
| Nuvvula/2016 (211) | India | Diagnosis | 4 | N | N | Y | Following the previous description  of Bader *et al.* |
| Khalesi/2016 (212) | Iran | Diagnosis | 15 | PRISMA | Y | Y | QUADAS |
| Marca/2020 (213) | Italy | Diagnosis | 66 | N | N | N | NR |
| Oliveira/2018 (214) | Brazil | Materials | 9 | PRISMA | N | Y | JADAD |
| Rodrigues/2019 (215) | Brazil | Materials | 14 | PRISMA and AMSTAR | Y | Y | CONSORT |
| Tardelli/2020 (216) | Brazil | Materials | 8 | N | N | N | NR |
| Goujat/2019 (217) | France | Materials | 23 | PRISMA | N | Y | Following previous description  of Onofre *et al.* |
| Ferle/2019 (218) | Germany | Materials | 115 | PRISMA | Y | N | NR |
| Moharrami/2019 (219) | Italy | Materials | 48 | PRISMA | N | N | NR |
| Snijder/2015 (220) | Netherlands | Materials | 53 | N | N | N | NR |
| Boersema/2016 (221) | Netherlands | Materials | 23 | PRISMA | N | N | NR |
| Vries/2020 (222) | Netherlands | Materials | 12 | PRISMA | N | N | NR |
| Oliveira/2019 (223) | Brazil | Methodology | 12 | PRISMA | Y | Y | QUADAS-2 |
| Pintor/2020 (224) | Brazil | Methodology | 40 | PRISMA | N | Y | ToxRTool |
| Ellis/2020 (225) | Canada | Methodology | 10 | N | Y | N | NR |
| Pisinger/2019 (226) | Denmark | Methodology | 94 | N | N | N | NR |
| Elshafay/2019 (227) | Egypt | Methodology | 65 | PRISMA | N | Y | PRISMA |
| Sinha/2017 (228) | India | Methodology | 61 | N | N | N | NR |
| Salamanna/2016 (229) | Italy | Methodology | 23 | PRISMA | N | N | NR |
| Maglio/2018 (230) | Italy | Methodology | 35 | PRISMA | N | N | NR |
| Kuik/2018 (231) | Netherlands | Methodology | 24 | PRISMA | N | Y | Developed by authors |
| Hoving/2019 (232) | Netherlands | Methodology | 42 | N | N | N | NR |
| Mai/2020 (233) | South Korea | Methodology | 10 | PRISMA | Y | Y | MINORS |
| Zhao/2018 (234) | UK | Methodology | 117 | PRISMA | N | Y | Developed by authors |
| Pasipanodya/2015 (235) | USA | Methodology | 22 | PRISMA | N | N | NR |
| Jiang/2018 (236) | China | Pharmacology | 56 | N | Y | N | NR |
| Bonczkowski/2016 (237) | Belgium | Pharmacology | 17 | N | N | N | NR |
| Fokou/2015 (238) | Ghana | Pharmacology | NR | N | N | N | NR |
| Ilango/2015 (239) | India | Pharmacology | NR | N | N | N | NR |
| Baumeister/2016 (240) | UK | Pharmacology | 17 | N | N | N | NR |
| Ling/2020 (241) | China | Toxicity | 59 | N | Y | Y | ToxRTool |
| Charles/2018 (242) | France | Toxicity | 36 | N | N | N | NR |
| Pronin/2018 (243) | Scotland | Toxicity | 16 | PRISMA | Y | N | NR |
| Marigliani/2020 (244) | USA | Toxicity | 83 | N | N | N | NR |

**References**

1. Strauss FJ, Nasirzade J, Kargarpoor Z, Stahli A, Gruber R. Effect of platelet-rich fibrin on cell proliferation, migration, differentiation, inflammation, and osteoclastogenesis: a systematic review of in vitro studies. Clin Oral Investig. 2020;24(2):569-84.

2. Magrin GL, Strauss FJ, Benfatti CAM, Maia LC, Gruber R. Effects of Short-Chain Fatty Acids on Human Oral Epithelial Cells and the Potential Impact on Periodontal Disease: A Systematic Review of In Vitro Studies. Int J Mol Sci. 2020;21(14).

3. Pavan LM, Rego DF, Elias ST, De Luca Canto G, Guerra EN. In vitro Anti-Tumor Effects of Statins on Head and Neck Squamous Cell Carcinoma: A Systematic Review. PLoS One. 2015;10(6):e0130476.

4. Diefenbach AL, Muniz F, Oballe HJR, Rosing CK. Antimicrobial activity of copaiba oil (Copaifera ssp.) on oral pathogens: Systematic review. Phytother Res. 2018;32(4):586-96.

5. Rego DF, Elias ST, Amato AA, Canto GL, Guerra EN. Anti-tumor effects of metformin on head and neck carcinoma cell lines: A systematic review. Oncol Lett. 2017;13(2):554-66.

6. Oliveira LV, Maia TS, Zancope K, Menezes MS, Soares CJ, Moura CCG. Can intra-radicular cleaning protocols increase the retention of fiberglass posts? A systematic review. Braz Oral Res. 2018;32:e16.

7. da Silva Hellwig AH, Heidrich D, Zanette RA, Scroferneker ML. In vitro susceptibility of chromoblastomycosis agents to antifungal drugs: A systematic review. J Glob Antimicrob Resist. 2019;16:108-14.

8. de Abreu PTR, de Arruda JAA, Mesquita RA, Abreu LG, Diniz IMA, Silva TA. Photobiomodulation effects on keratinocytes cultured in vitro: a critical review. Lasers Med Sci. 2019;34(9):1725-34.

9. da Silva JL, Silva-de-Oliveira AFS, Andraus RAC, Maia LP. Effects of low level laser therapy in cancer cells-a systematic review of the literature. Lasers Med Sci. 2020;35(3):523-9.

10. Picco DCR, Cavalcante LLR, Trevisan RLB, Souza-Gabriel AE, Borsatto MC, Corona SAM. Effect of curcumin-mediated photodynamic therapy on Streptococcus mutans and Candida albicans: A systematic review of in vitro studies. Photodiagnosis Photodyn Ther. 2019;27:455-61.

11. Sanches PL, Geaquinto LRO, Cruz R, Schuck DC, Lorencini M, Granjeiro JM, et al. Toxicity Evaluation of TiO2 Nanoparticles on the 3D Skin Model: A Systematic Review. Front Bioeng Biotechnol. 2020;8:575.

12. Xiao Z, Li C, Shan J, Luo L, Feng L, Lu J, et al. Mechanisms of renal cell apoptosis induced by cyclosporine A: a systematic review of in vitro studies. Am J Nephrol. 2011;33(6):558-66.

13. Ni W, Shao X, Di X, Cui J, Wang R, Liu Y. In vitro synergy of polymyxins with other antibiotics for Acinetobacter baumannii: a systematic review and meta-analysis. Int J Antimicrob Agents. 2015;45(1):8-18.

14. Asweto CO, Wu J, Alzain MA, Hu H, Andrea S, Feng L, et al. Cellular pathways involved in silica nanoparticles induced apoptosis: A systematic review of in vitro studies. Environ Toxicol Pharmacol. 2017;56:191-7.

15. Li J, Yang X, Chen L, Duan X, Jiang Z. In Vitro Activity of Various Antibiotics in Combination with Tigecycline Against Acinetobacter baumannii: A Systematic Review and Meta-Analysis. Microbial drug resistance (Larchmont, NY). 2017;23(8):982-93.

16. Simundic Munitic M, Poklepovic Pericic T, Utrobicic A, Bago I, Puljak L. Antimicrobial efficacy of commercially available endodontic bioceramic root canal sealers: A systematic review. PLoS One. 2019;14(10):e0223575.

17. Saravanan M, Vahidi H, Medina Cruz D, Vernet-Crua A, Mostafavi E, Stelmach R, et al. Emerging Antineoplastic Biogenic Gold Nanomaterials for Breast Cancer Therapeutics: A Systematic Review. Int J Nanomedicine. 2020;15:3577-95.

18. Khanafer N, Daneman N, Greene T, Simor A, Vanhems P, Samore M, et al. Susceptibilities of clinical Clostridium difficile isolates to antimicrobials: a systematic review and meta-analysis of studies since 1970. Clin Microbiol Infect. 2018;24(2):110-7.

19. Pummer A, Knuttel H, Hiller KA, Buchalla W, Cieplik F, Maisch T. Antimicrobial efficacy of irradiation with visible light on oral bacteria in vitro: a systematic review. Future Med Chem. 2017;9(13):1557-74.

20. Raju K L, Augustine D, Rao RS, S V S, Haragannavar VC, Nambiar S, et al. Biomarkers in Tumorigenesis Using Cancer Cell Lines: A Systematic Review. Asian Pac J Cancer Prev. 2017;18(9):2329-37.

21. Moradi MT, Rafieian-Kopaei M, Karimi A. A review study on the effect of Iranian herbal medicines against in vitro replication of herpes simplex virus. Avicenna J Phytomed. 2016;6(5):506-15.

22. Samiei M, Farjami A, Dizaj SM, Lotfipour F. Nanoparticles for antimicrobial purposes in Endodontics: A systematic review of in vitro studies. Mater Sci Eng C Mater Biol Appl. 2016;58:1269-78.

23. Hasannejad-Bibalan M, Mojtahedi A, Biglari H, Halaji M, Sedigh Ebrahim-Saraie H. Antibacterial Activity of Tedizolid, a Novel Oxazolidinone Against Methicillin-Resistant Staphylococcus aureus: A Systematic Review and Meta-Analysis. Microbial drug resistance (Larchmont, NY). 2019;25(9):1330-7.

24. Mansourian M, Marateb HR, Vaseghi G. The effect of extremely low-frequency magnetic field (50-60 Hz) exposure on spontaneous apoptosis: The results of a meta-analysis. Adv Biomed Res. 2016;5:141-.

25. Khaledi A, Meskini M. A Systematic Review of the Effects of Satureja Khuzestanica Jamzad and Zataria Multiflora Boiss against Pseudomonas Aeruginosa. Iran J Med Sci. 2020;45(2):83-90.

26. Zusman O, Avni T, Leibovici L, Adler A, Friberg L, Stergiopoulou T, et al. Systematic review and meta-analysis of in vitro synergy of polymyxins and carbapenems. Antimicrob Agents Chemother. 2013;57(10):5104-11.

27. Gizzo S, Noventa M, Di Gangi S, Litta P, Saccardi C, D'Antona D, et al. Could in-vitro studies on Ishikawa cell lines explain the endometrial safety of raloxifene? Systematic literature review and starting points for future oncological research. European journal of cancer prevention : the official journal of the European Cancer Prevention Organisation (ECP). 2015;24(6):497-507.

28. Gianfredi V, Nucci D, Vannini S, Villarini M, Moretti M. In vitro Biological Effects of Sulforaphane (SFN), Epigallocatechin-3-gallate (EGCG), and Curcumin on Breast Cancer Cells: A Systematic Review of the Literature. Nutr Cancer. 2017;69(7):969-78.

29. Gianfredi V, Vannini S, Moretti M, Villarini M, Bragazzi NL, Izzotti A, et al. Sulforaphane and Epigallocatechin Gallate Restore Estrogen Receptor Expression by Modulating Epigenetic Events in the Breast Cancer Cell Line MDA-MB-231: A Systematic Review and Meta-Analysis. J Nutrigenet Nutrigenomics. 2017;10(3-4):126-35.

30. Zhurakivska K, Troiano G, Caponio VCA, Dioguardi M, Arena C, Lo Muzio L. The Effects of Adjuvant Fermented Wheat Germ Extract on Cancer Cell Lines: A Systematic Review. Nutrients. 2018;10(10).

31. Lemma MT, Ahmed AM, Elhady MT, Ngo HT, Vu TL, Sang TK, et al. Medicinal plants for in vitro antiplasmodial activities: A systematic review of literature. Parasitol Int. 2017;66(6):713-20.

32. Puidokas T, Kubilius M, Stumbras A, Juodzbalys G. Effect of leukocytes included in platelet concentrates on cell behaviour. Platelets. 2019;30(8):937-45.

33. Almoudi MM, Hussein AS, Abu Hassan MI, Mohamad Zain N. A systematic review on antibacterial activity of zinc against Streptococcus mutans. Saudi Dent J. 2018;30(4):283-91.

34. Lim BSH, Parolia A, Chia MSY, Jayaraman J, Nagendrababu V. Antimicrobial efficacy of QMix on Enterococcus faecalis infected root canals: a systematic review of in vitro studies. Restor Dent Endod. 2020;45(2):e23.

35. Hlashwayo DF, Barbosa F, Langa S, Sigauque B, Bila CG. A Systematic Review of In Vitro Activity of Medicinal Plants from Sub-Saharan Africa against Campylobacter spp. Evid Based Complement Alternat Med. 2020;2020:9485364.

36. Montano M, Bakker EJ, Murk AJ. Meta-analysis of supramaximal effects in in vitro estrogenicity assays. Toxicol Sci. 2010;115(2):462-74.

37. Golbach LA, Portelli LA, Savelkoul HF, Terwel SR, Kuster N, de Vries RB, et al. Calcium homeostasis and low-frequency magnetic and electric field exposure: A systematic review and meta-analysis of in vitro studies. Environ Int. 2016;92-93:695-706.

38. Dobbenga S, Fratila-Apachitei LE, Zadpoor AA. Nanopattern-induced osteogenic differentiation of stem cells - A systematic review. Acta Biomater. 2016;46:3-14.

39. Hoppenbrouwers T, Autar ASA, Sultan AR, Abraham TE, van Cappellen WA, Houtsmuller AB, et al. In vitro induction of NETosis: Comprehensive live imaging comparison and systematic review. PLoS One. 2017;12(5):e0176472.

40. Pardo-Aldave K, Pareja-Vásquez M, Guillén A, Ureta-Tapia JM. Actividad antimicrobiana in vitro del camu camu (Myrciaria dubia) contra microorganismos orales: una revisión sistemática. Revista Peruana de Medicina Experimental y Salud Publica. 2019;36:573-82.

41. AlShwaimi E, Bogari D, Ajaj R, Al-Shahrani S, Almas K, Majeed A. In Vitro Antimicrobial Effectiveness of Root Canal Sealers against Enterococcus faecalis: A Systematic Review. J Endod. 2016;42(11):1588-97.

42. Alasqah MN. Antimicrobial efficacy of photodynamic therapy on dental implant surfaces: A systematic review of in vitro studies. Photodiagnosis Photodyn Ther. 2019;25:349-53.

43. Al-Hamdan RS. Does bond integrity of bleached enamel increases with phototherapy? A systematic review. Photodiagnosis Photodyn Ther. 2019;25:401-5.

44. Batista Napotnik T, Reberšek M, Vernier PT, Mali B, Miklavčič D. Effects of high voltage nanosecond electric pulses on eukaryotic cells (in vitro): A systematic review. Bioelectrochemistry. 2016;110:1-12.

45. Kaufmann M, Lenherr P, Walter C, Thurnheer T, Attin T, Wiedemeier DB, et al. Comparing the Antimicrobial In Vitro Efficacy of Amoxicillin/Metronidazole against Azithromycin-A Systematic Review. Dent J (Basel). 2018;6(4).

46. Kumar R, Griffin M, Adigbli G, Kalavrezos N, Butler PE. Lipotransfer for radiation-induced skin fibrosis. Br J Surg. 2016;103(8):950-61.

47. Jabran A, Peach C, Ren L. Biomechanical analysis of plate systems for proximal humerus fractures: a systematic literature review. Biomed Eng Online. 2018;17(1):47.

48. Holliday RS, Campbell J, Preshaw PM. Effect of nicotine on human gingival, periodontal ligament and oral epithelial cells. A systematic review of the literature. J Dent. 2019;86:81-8.

49. Scott RE, Greenwood SL, Hayes DJL, Baker BC, Jones RL, Heazell AEP. Effects of hydroxychloroquine on the human placenta-Findings from in vitro experimental data and a systematic review. Reprod Toxicol. 2019;87:50-9.

50. Prpa EJ, Bajka BH, Ellis PR, Butterworth PJ, Corpe CP, Hall WL. A systematic review of in vitro studies evaluating the inhibitory effects of polyphenol-rich fruit extracts on carbohydrate digestive enzymes activity: a focus on culinary fruits consumed in Europe. Crit Rev Food Sci Nutr. 2020:1-21.

51. Ting M, Whitaker EJ, Albandar JM. Systematic review of the in vitro effects of statins on oral and perioral microorganisms. Eur J Oral Sci. 2016;124(1):4-10.

52. Chemaly RF, Hill JA, Voigt S, Peggs KS. In vitro comparison of currently available and investigational antiviral agents against pathogenic human double-stranded DNA viruses: A systematic literature review. Antiviral Res. 2019;163:50-8.

53. Tam DNH, Tawfik GM, El-Qushayri AE, Mehyar GM, Istanbuly S, Karimzadeh S, et al. Correlation between anti-malarial and anti-haemozoin activities of anti-malarial compounds. Malaria Journal. 2020;19(1):298.

54. Liu Y, Eaton ED, Wills TE, McCann SK, Antonic A, Howells DW. Human Ischaemic Cascade Studies Using SH-SY5Y Cells: a Systematic Review and Meta-Analysis. Transl Stroke Res. 2018;9(6):564-74.

55. Lemos NE, de Almeida Brondani L, Dieter C, Rheinheimer J, Bouças AP, Bauermann Leitão C, et al. Use of additives, scaffolds and extracellular matrix components for improvement of human pancreatic islet outcomes in vitro: A systematic review. Islets. 2017;9(5):73-86.

56. Pacheco CMR, Ferreira PE, Sacaki CS, Tannous LA, Zotarelli-Filho IJ, Guarita-Souza LC, et al. In vitro differentiation capacity of human breastmilk stem cells: A systematic review. World J Stem Cells. 2019;11(11):1005-19.

57. Chierrito D, Villas-Boas CB, Tonin FS, Fernandez-Llimos F, Sanches ACC, de Mello JCP. Using Cell Cultures for the Investigation of Treatments for Attention Deficit Hyperactivity Disorder: A Systematic Review. Current neuropharmacology. 2019;17(10):916-25.

58. Giorgi Silveira R, Perelló Ferrúa C, do Amaral CC, Fernandez Garcia T, de Souza KB, Nedel F. MicroRNAs expressed in neuronal differentiation and their associated pathways: Systematic review and bioinformatics analysis. Brain research bulletin. 2020;157:140-8.

59. Rahman NA, Rasil A, Meyding-Lamade U, Craemer EM, Diah S, Tuah AA, et al. Immortalized endothelial cell lines for in vitro blood-brain barrier models: A systematic review. Brain Res. 2016;1642:532-45.

60. Ren C, McGrath C, Jin L, Zhang C, Yang Y. Effect of diode low-level lasers on fibroblasts derived from human periodontal tissue: a systematic review of in vitro studies. Lasers Med Sci. 2016;31(7):1493-510.

61. Li M, Zhang C, Yang Y. Effects of mechanical forces on osteogenesis and osteoclastogenesis in human periodontal ligament fibroblasts: A systematic review of in vitro studies. Bone Joint Res. 2019;8(1):19-31.

62. Kirkegaard T, Gögenur M, Gögenur I. Assessment of perioperative stress in colorectal cancer by use of in vitro cell models: a systematic review. PeerJ. 2017;5:e4033.

63. Bleuel J, Zaucke F, Bruggemann GP, Niehoff A. Effects of cyclic tensile strain on chondrocyte metabolism: a systematic review. PLoS One. 2015;10(3):e0119816.

64. Dissemond J, Steinmann J, Munter KC, Brill H, Bottrich JG, Braunwarth H, et al. Risk and clinical impact of bacterial resistance/susceptibility to silver-based wound dressings: a systematic review. J Wound Care. 2020;29(4):221-34.

65. Jayanegara A, Sujarnoko TUP, Ridla M, Kondo M, Kreuzer M. Silage quality as influenced by concentration and type of tannins present in the material ensiled: A meta-analysis. Journal of animal physiology and animal nutrition. 2019;103(2):456-65.

66. Tabatabaei-Malazy O, Larijani B, Abdollahi M. A systematic review of in vitro studies conducted on effect of herbal products on secretion of insulin from Langerhans islets. J Pharm Pharm Sci. 2012;15(3):447-66.

67. Mohammadrezaei D, Golzar H, Rezai Rad M, Omidi M, Rashedi H, Yazdian F, et al. In vitro effect of graphene structures as an osteoinductive factor in bone tissue engineering: A systematic review. Journal of biomedical materials research Part A. 2018;106(8):2284-343.

68. Franzoni M, Walsh MT. Towards the Identification of Hemodynamic Parameters Involved in Arteriovenous Fistula Maturation and Failure: A Review. Cardiovascular Engineering and Technology. 2017;8(3):342-56.

69. Rotelli MT, Bocale D, De Fazio M, Ancona P, Scalera I, Memeo R, et al. IN-VITRO evidence for the protective properties of the main components of the Mediterranean diet against colorectal cancer: A systematic review. Surgical oncology. 2015;24(3):145-52.

70. Maccarana L, Cattani M, Tagliapietra F, Schiavon S, Bailoni L, Mantovani R. Methodological factors affecting gas and methane production during in vitro rumen fermentation evaluated by meta-analysis approach. J Anim Sci Biotechnol. 2016;7:35.

71. Veronesi F, Tschon M, Visani A, Fini M. Biosensors for real-time monitoring of physiological processes in the musculoskeletal system: A systematic review. J Cell Physiol. 2019;234(12):21504-18.

72. Gentile P, Sterodimas A, Pizzicannella J, Dionisi L, De Fazio D, Calabrese C, et al. Systematic Review: Allogenic Use of Stromal Vascular Fraction (SVF) and Decellularized Extracellular Matrices (ECM) as Advanced Therapy Medicinal Products (ATMP) in Tissue Regeneration. Int J Mol Sci. 2020;21(14).

73. Contreras-Ochoa CO, Lagunas-Martinez A, Belkind-Gerson J, Correa D. Toxoplasma gondii invasion and replication in astrocyte primary cultures and astrocytoma cell lines: systematic review of the literature. Parasitol Res. 2012;110(6):2089-94.

74. Peplow PV, Chatterjee MP. A review of the influence of growth factors and cytokines in in vitro human keratinocyte migration. Cytokine. 2013;62(1):1-21.

75. Tong M, Viall CA, Chamley LW. Antiphospholipid antibodies and the placenta: a systematic review of their in vitro effects and modulation by treatment. Human reproduction update. 2015;21(1):97-118.

76. Vasyutin I, Zerihun L, Ivan C, Atala A. Bladder Organoids and Spheroids: Potential Tools for Normal and Diseased Tissue Modelling. Anticancer Res. 2019;39(3):1105-18.

77. Garcia-Sanz V, Paredes-Gallardo V, Mendoza-Yero O, Carbonell-Leal M, Albaladejo A, Montiel-Company JM, et al. The effects of lasers on bond strength to ceramic materials: A systematic review and meta-analysis. PLoS One. 2018;13(1):e0190736.

78. Bryant C, Barnett J. Consumer acceptance of cultured meat: A systematic review. Meat Sci. 2018;143:8-17.

79. Chew D, Green V, Riley A, England RJ, Greenman J. The Changing Face of in vitro Culture Models for Thyroid Cancer Research: A Systematic Literature Review. Frontiers in Surgery. 2020;7(43).

80. Bahsoun S, Coopman K, Akam EC. The impact of cryopreservation on bone marrow-derived mesenchymal stem cells: a systematic review. J Transl Med. 2019;17(1):397.

81. Dalton B, Whitmore V, Patsalos O, Ibrahim MAA, Schmidt U, Himmerich H. A systematic review of in vitro cytokine production in eating disorders. Mol Cell Endocrinol. 2019;497:110308.

82. Arilla FV, Yeung M, Bell K, Rahnemai-Azar AA, Rothrauff BB, Fu FH, et al. Experimental Execution of the Simulated Pivot-Shift Test: A Systematic Review of Techniques. Arthroscopy. 2015;31(12):2445-54 e2.

83. Bates NA, Myer GD, Shearn JT, Hewett TE. Anterior cruciate ligament biomechanics during robotic and mechanical simulations of physiologic and clinical motion tasks: a systematic review and meta-analysis. Clin Biomech (Bristol, Avon). 2015;30(1):1-13.

84. Slette EL, Mikula JD, Schon JM, Marchetti DC, Kheir MM, Turnbull TL, et al. Biomechanical Results of Lateral Extra-articular Tenodesis Procedures of the Knee: A Systematic Review. Arthroscopy. 2016;32(12):2592-611.

85. Nawafleh N, Hatamleh M, Elshiyab S, Mack F. Lithium Disilicate Restorations Fatigue Testing Parameters: A Systematic Review. J Prosthodont. 2016;25(2):116-26.

86. Shahmiri R, Standard OC, Hart JN, Sorrell CC. Optical properties of zirconia ceramics for esthetic dental restorations: A systematic review. J Prosthet Dent. 2018;119(1):36-46.

87. Elshiyab SH, Nawafleh N, George R. Survival and testing parameters of zirconia-based crowns under cyclic loading in an aqueous environment: A systematic review. J Investig Clin Dent. 2017;8(4).

88. Thammajaruk P, Inokoshi M, Chong S, Guazzato M. Bonding of composite cements to zirconia: A systematic review and meta-analysis of in vitro studies. J Mech Behav Biomed Mater. 2018;80:258-68.

89. Kulkarni S, Meer M, George R. The effect of photobiomodulation on human dental pulp-derived stem cells: systematic review. Lasers Med Sci. 2020;35(9):1889-97.

90. Schmid-Schwap M, Graf A, Preinerstorfer A, Watts DC, Piehslinger E, Schedle A. Microleakage after thermocycling of cemented crowns--a meta-analysis. Dent Mater. 2011;27(9):855-69.

91. Wehner C, Lettner S, Moritz A, Andrukhov O, Rausch-Fan X. Effect of bisphosphonate treatment of titanium surfaces on alkaline phosphatase activity in osteoblasts: a systematic review and meta-analysis. BMC Oral Health. 2020;20(1):125.

92. Swimberghe RCD, Coenye T, De Moor RJG, Meire MA. Biofilm model systems for root canal disinfection: a literature review. Int Endod J. 2019;52(5):604-28.

93. Osmanovic A, Halilovic S, Kurtovic-Kozaric A, Hadziabdic N. Evaluation of periodontal ligament cell viability in different storage media based on human PDL cell culture experiments-A systematic review. Dent Traumatol. 2018;34(6):384-93.

94. Chaves CA, Machado AL, Vergani CE, de Souza RF, Giampaolo ET. Cytotoxicity of denture base and hard chairside reline materials: a systematic review. J Prosthet Dent. 2012;107(2):114-27.

95. da Costa DC, Coutinho M, de Sousa AS, Ennes JP. A meta-analysis of the most indicated preparation design for porcelain laminate veneers. The journal of adhesive dentistry. 2013;15(3):215-20.

96. Sarkis-Onofre R, Skupien JA, Cenci MS, Moraes RR, Pereira-Cenci T. The role of resin cement on bond strength of glass-fiber posts luted into root canals: a systematic review and meta-analysis of in vitro studies. Oper Dent. 2014;39(1):E31-44.

97. Bernades Kde O, Hilgert LA, Ribeiro AP, Garcia FC, Pereira PN. The influence of hemostatic agents on dentin and enamel surfaces and dental bonding: a systematic review. J Am Dent Assoc. 2014;145(11):1120-8.

98. Kaizer MR, de Oliveira-Ogliari A, Cenci MS, Opdam NJM, Moraes RR. Do nanofill or submicron composites show improved smoothness and gloss? A systematic review of in vitro studies. Dental Materials. 2014;30(4):e41-e78.

99. Moraes AP, Sarkis-Onofre R, Moraes RR, Cenci MS, Soares CJ, Pereira-Cenci T. Can Silanization Increase the Retention of Glass-fiber posts? A Systematic Review and Meta-analysis of In Vitro Studies. Oper Dent. 2015;40(6):567-80.

100. Rosa WL, Piva E, Silva AF. Bond strength of universal adhesives: A systematic review and meta-analysis. J Dent. 2015;43(7):765-76.

101. Pereira GKR, Venturini AB, Silvestri T, Dapieve KS, Montagner AF, Soares FZM, et al. Low-temperature degradation of Y-TZP ceramics: A systematic review and meta-analysis. J Mech Behav Biomed Mater. 2015;55:151-63.

102. Skupien JA, Sarkis-Onofre R, Cenci MS, Moraes RR, Pereira-Cenci T. A systematic review of factors associated with the retention of glass fiber posts. Braz Oral Res. 2015;29.

103. Altmann AS, Collares FM, Leitune VC, Samuel SM. The effect of antimicrobial agents on bond strength of orthodontic adhesives: a meta-analysis of in vitro studies. Orthod Craniofac Res. 2016;19(1):1-9.

104. Soares FZ, Follak A, da Rosa LS, Montagner AF, Lenzi TL, Rocha RO. Bovine tooth is a substitute for human tooth on bond strength studies: A systematic review and meta-analysis of in vitro studies. Dent Mater. 2016;32(11):1385-93.

105. Aurélio IL, Marchionatti AME, Montagner AF, May LG, Soares FZM. Does air particle abrasion affect the flexural strength and phase transformation of Y-TZP? A systematic review and meta-analysis. Dental Materials. 2016;32(6):827-45.

106. Lenzi TL, Gimenez T, Tedesco TK, Mendes FM, Rocha Rde O, Raggio DP. Adhesive systems for restoring primary teeth: a systematic review and meta-analysis of in vitro studies. Int J Paediatr Dent. 2016;26(5):364-75.

107. Rosa MI, Schambeck VS, Dondossola ER, Alexandre MC, Tuon L, Grande AJ, et al. Laser fluorescence of caries detection in permanent teeth in vitro: a systematic review and meta-analysis. J Evid Based Med. 2016;9(4):213-24.

108. Reis AF, Vestphal M, Amaral RCD, Rodrigues JA, Roulet JF, Roscoe MG. Efficiency of polymerization of bulk-fill composite resins: a systematic review. Braz Oral Res. 2017;31(suppl 1):e59.

109. Ferrúa CP, Centeno EGZ, Rosa LCD, Amaral CCD, Severo RF, Sarkis-Onofre R, et al. How has dental pulp stem cells isolation been conducted? A scoping review. Braz Oral Res. 2017;31:e87.

110. Silva E, Rover G, Belladonna FG, De-Deus G, da Silveira Teixeira C, da Silva Fidalgo TK. Impact of contracted endodontic cavities on fracture resistance of endodontically treated teeth: a systematic review of in vitro studies. Clin Oral Investig. 2018;22(1):109-18.

111. Maske TT, van de Sande FH, Arthur RA, Huysmans M, Cenci MS. In vitro biofilm models to study dental caries: a systematic review. Biofouling. 2017;33(8):661-75.

112. Pardal-Pelaez B, Montero J. Preload loss of abutment screws after dynamic fatigue in single implant-supported restorations. A systematic review. J Clin Exp Dent. 2017;9(11):e1355-e61.

113. de Mello CC, Bitencourt SB, Dos Santos DM, Pesqueira AA, Pellizzer EP, Goiato MC. The Effect of Surface Treatment on Shear Bond Strength between Y-TZP and Veneer Ceramic: A Systematic Review and Meta-Analysis. J Prosthodont. 2018;27(7):624-35.

114. Corrêa G, Brondani LP, Sarkis-Onofre R, Bergoli C. Restorative strategies for weakened roots: Systematic review and Meta-analysis of in vitro studies. Brazilian dental science. 2019;22:124-34.

115. Mello CC, Lemos CAA, de Luna Gomes JM, Verri FR, Pellizzer EP. CAD/CAM vs Conventional Technique for Fabrication of Implant-Supported Frameworks: A Systematic Review and Meta-analysis of In Vitro Studies. Int J Prosthodont. 2019;32(2):182-92.

116. Perroni AP, Kaizer MR, Della Bona A, Moraes RR, Boscato N. Influence of light-cured luting agents and associated factors on the color of ceramic laminate veneers: A systematic review of in vitro studies. Dent Mater. 2018;34(11):1610-24.

117. de Carvalho MFF, Leijôto-Lannes ACN, Rodrigues MCN, Nogueira LC, Ferraz NKL, Moreira AN, et al. Viability of Bovine Teeth as a Substrate in Bond Strength Tests: A Systematic Review and Meta-analysis. The journal of adhesive dentistry. 2018;20(6):471-9.

118. Caldas IP, Alves GG, Barbosa IB, Scelza P, de Noronha F, Scelza MZ. In vitro cytotoxicity of dental adhesives: A systematic review. Dent Mater. 2019;35(2):195-205.

119. Menezes-Silva R, Cabral RN, Pascotto RC, Borges AFS, Martins CC, Navarro MFL, et al. Mechanical and optical properties of conventional restorative glass-ionomer cements - a systematic review. J Appl Oral Sci. 2019;27:e2018357.

120. Marchionatti AME, Aurélio IL, May LG. Does veneering technique affect the flexural strength or load-to-failure of bilayer Y-TZP? A systematic review and meta-analysis. The Journal of Prosthetic Dentistry. 2018;119(6):916-24.

121. Bohrer TC, Fontana PE, Lenzi TL, Soares FZM, Rocha RdO. Can Endodontic Irrigating Solutions Influence the Bond Strength of Adhesives to Coronal Dental Substrates? A Systematic Review and Meta-Analysis of In Vitro Studies. The journal of adhesive dentistry. 2018;20(6):481-94.

122. Schestatsky R, Dartora G, Felberg R, Spazzin AO, Sarkis-Onofre R, Bacchi A, et al. Do endodontic retreatment techniques influence the fracture strength of endodontically treated teeth? A systematic review and meta-analysis. J Mech Behav Biomed Mater. 2019;90:306-12.

123. de Almeida CM, da Rosa WLO, Meereis CTW, de Almeida SM, Ribeiro JS, da Silva AF, et al. Efficacy of antimicrobial agents incorporated in orthodontic bonding systems: a systematic review and meta-analysis. J Orthod. 2018;45(2):79-93.

124. Martins FV, Vasques WF, Fonseca EM. How the Variations of the Thickness in Ceramic Restorations of Lithium Disilicate and the Use of Different Photopolymerizers Influence the Degree of Conversion of the Resin Cements: A Systematic Review and Meta-Analysis. Journal of Prosthodontics. 2019;28(1):e395-e403.

125. Martins FV, Vasques WF, Fonseca EM. Evaluation of the efficiency of fluoride-releasing adhesives for preventing secondary caries in-vitro: a systematic review and meta-analysis. Eur Arch Paediatr Dent. 2019;20(1):1-8.

126. de Sousa A, Franca K, de Lucas Rezende LVM, do Nascimento Poubel DL, Almeida JCF, de Toledo IP, et al. In vitro tooth reattachment techniques: A systematic review. Dent Traumatol. 2018;34(5):297-310.

127. Munchow EA, Meereis CTW, de Oliveira da Rosa WL, da Silva AF, Piva E. Polymerization shrinkage stress of resin-based dental materials: A systematic review and meta-analyses of technique protocol and photo-activation strategies. J Mech Behav Biomed Mater. 2018;82:77-86.

128. Pires CW, Soldera EB, Bonzanini LIL, Lenzi TL, Soares FZM, Montagner AF, et al. Is Adhesive Bond Strength Similar in Primary and Permanent Teeth? A Systematic Review and Meta-analysis. The journal of adhesive dentistry. 2018;20(2):87-97.

129. Fumes AC, da Silva Telles PD, Corona SAM, Borsatto MC. Effect of aPDT on Streptococcus mutans and Candida albicans present in the dental biofilm: Systematic review. Photodiagnosis Photodyn Ther. 2018;21:363-6.

130. Cury SE, Aliaga-Del Castillo A, Pinzan A, Sakoda KL, Bellini-Pereira SA, Janson G. Orthodontic brackets friction changes after clinical use: A systematic review. J Clin Exp Dent. 2019;11(5):e482-e90.

131. Tavares SJO, Sarmento EB, Guimaraes LDS, Antunes LAA, Antunes LS, Gomes CC. The influence of kinematics of engine-driven nickel-titanium instruments on root canal shape assessed by micro-computed tomography: a systematic review. Acta Odontol Scand. 2019;77(5):347-58.

132. Resende KKM, Faria GP, Longo DL, Martins LJO, Costa CRR. In vitro evaluation of plants as storage media for avulsed teeth: A systematic review. Dent Traumatol. 2020;36(1):3-18.

133. Nogueira I, de Oliveira PF, Magno M, Ferreira D, Maia L, Rabello T. Does the application of an adhesive layer improve the bond strength of etched and silanized glass ceramics to resin-based materials? A systematic review and meta-analysis. The Journal of Prosthetic Dentistry. 2020.

134. Miranda ML, Silva BNS, Salomao KB, de Oliveira AB, Gabbai-Armelin PR, Brighenti FL. Effect of arginine on microorganisms involved in dental caries: a systematic literature review of in vitro studies. Biofouling. 2020;36(6):696-709.

135. Fonseca JM, Troconis CC, Palmier NR, Gomes-Silva W, Paglioni MD, Araujo AL, et al. The impact of head and neck radiotherapy on the dentine-enamel junction: a systematic review. Med Oral Patol Oral Cir Bucal. 2020;25(1):e96-e105.

136. Leao RS, Moraes SLD, Gomes JML, Lemos CAA, Casado B, Vasconcelos B, et al. Influence of addition of zirconia on PMMA: A systematic review. Mater Sci Eng C Mater Biol Appl. 2020;106:110292.

137. Brandeburski SBN, Vidal ML, Collares K, Zhang Y, Della Bona A. Edge chipping test in dentistry: A comprehensive review. Dent Mater. 2020;36(3):e74-e84.

138. Kreve S, Candido Dos Reis A. Influence of the electrostatic condition of the titanium surface on bacterial adhesion: A systematic review. J Prosthet Dent. 2020.

139. Ehsani S, Mandich MA, El-Bialy TH, Flores-Mir C. Frictional resistance in self-ligating orthodontic brackets and conventionally ligated brackets. A systematic review. Angle Orthod. 2009;79(3):592-601.

140. Archambault A, Lacoursiere R, Badawi H, Major PW, Carey J, Flores-Mir C. Torque expression in stainless steel orthodontic brackets. A systematic review. Angle Orthod. 2010;80(1):201-10.

141. Nassar U, Aziz T, Flores-Mir C. Dimensional stability of irreversible hydrocolloid impression materials as a function of pouring time: a systematic review. J Prosthet Dent. 2011;106(2):126-33.

142. Passos SP, Torrealba Y, Major P, Linke B, Flores-Mir C, Nychka JA. In vitro wear behavior of zirconia opposing enamel: a systematic review. J Prosthodont. 2014;23(8):593-601.

143. Tan M, Chai Z, Sun C, Hu B, Gao X, Chen Y, et al. Comparative evaluation of the vertical fracture resistance of endodontically treated roots filled with Gutta-percha and Resilon: a meta-analysis of in vitro studies. BMC Oral Health. 2018;18(1):107.

144. Yu H, Ozcan M, Yoshida K, Cheng H, Sawase T. Bonding to industrial indirect composite blocks: A systematic review and meta-analysis. Dent Mater. 2020;36(1):119-34.

145. Yu H, Chen YH, Cheng H, Sawase T. Finish-line designs for ceramic crowns: A systematic review and meta-analysis. J Prosthet Dent. 2019;122(1):22-30 e5.

146. Wang C, Shi YF, Xie PJ, Wu JH. Accuracy of digital complete dentures: A systematic review of in vitro studies. J Prosthet Dent. 2020.

147. Razdan A, Benetti AR, Bjørndal L. Do in vitro solubility studies on endodontic sealers demonstrate a high level of evidence? A systematic review. Acta Odontologica Scandinavica. 2019;77(4):253-63.

148. Elkaffas AA, Eltoukhy RI, Elnegoly SA, Mahmoud SH. The effect of preheating resin composites on surface hardness: a systematic review and meta-analysis. Restor Dent Endod. 2019;44(4):e41.

149. Janjic M, Docheva D, Trickovic Janjic O, Wichelhaus A, Baumert U. In Vitro Weight-Loaded Cell Models for Understanding Mechanodependent Molecular Pathways Involved in Orthodontic Tooth Movement: A Systematic Review. Stem Cells Int. 2018;2018:3208285.

150. Herbst SR, Krois J, Schwendicke F. Comparator Choice in Studies Testing Endodontic Instrument Fatigue Resistance: A Network Analysis. J Endod. 2019;45(6):784-90.

151. Mustafa HA, Soares AP, Paris S, Elhennawy K, Zaslansky P. The forgotten merits of GIC restorations: a systematic review. Clin Oral Investig. 2020;24(7):2189-201.

152. Bethke A, Pieralli S, Kohal RJ, Burkhardt F, von Stein-Lausnitz M, Vach K, et al. Fracture Resistance of Zirconia Oral Implants In Vitro: A Systematic Review and Meta-Analysis. Materials (Basel). 2020;13(3).

153. Tzanakakis EG, Tzoutzas IG, Koidis PT. Is there a potential for durable adhesion to zirconia restorations? A systematic review. J Prosthet Dent. 2016;115(1):9-19.

154. Iliadi A, Koletsi D, Eliades T. Forces and moments generated by aligner-type appliances for orthodontic tooth movement: A systematic review and meta-analysis. Orthod Craniofac Res. 2019;22(4):248-58.

155. Gizani S, Kloukos D, Papadimitriou A, Roumani T, Twetman S. Is Bleaching Effective in Managing Post-orthodontic White-spot Lesions? A Systematic Review. Oral health & preventive dentistry. 2020;18(1):1-10.

156. Solanki NP, Venkappa KK, Shah NC. Biocompatibility and sealing ability of mineral trioxide aggregate and biodentine as root-end filling material: A systematic review. J Conserv Dent. 2018;21(1):10-5.

157. Ajay R, Suma K, Ali SA. Monomer Modifications of Denture Base Acrylic Resin: A Systematic Review and Meta-analysis. J Pharm Bioallied Sci. 2019;11(Suppl 2):S112-S25.

158. Bangera MK, Kotian R, N R. Effect of titanium dioxide nanoparticle reinforcement on flexural strength of denture base resin: A systematic review and meta-analysis. Jpn Dent Sci Rev. 2020;56(1):68-76.

159. Parikh M, Kishan KV, Solanki NP, Parikh M, Savaliya K, Bindu VH, et al. Efficacy of Removal of Calcium Hydroxide Medicament from Root Canals by Endoactivator and Endovac Irrigation Techniques: A Systematic Review of In vitro Studies. Contemp Clin Dent. 2019;10(1):135-42.

160. Pandey P, Aggarwal H, Tikku AP, Singh A, Bains R, Mishra S. Comparative evaluation of sealing ability of gutta percha and resilon as root canal filling materials- a systematic review. J Oral Biol Craniofac Res. 2020;10(2):220-6.

161. Jayanegara A, Wina E, Takahashi J. Meta-analysis on Methane Mitigating Properties of Saponin-rich Sources in the Rumen: Influence of Addition Levels and Plant Sources. Asian-Australas J Anim Sci. 2014;27(10):1426-35.

162. Shahravan A, Haghdoost AA, Adl A, Rahimi H, Shadifar F. Effect of smear layer on sealing ability of canal obturation: a systematic review and meta-analysis. J Endod. 2007;33(2):96-105.

163. Motamedian SR, Hosseinpour S, Ahsaie MG, Khojasteh A. Smart scaffolds in bone tissue engineering: A systematic review of literature. World J Stem Cells. 2015;7(3):657-68.

164. Hindy A, Farahmand F, Tabatabaei FS. In vitro biological outcome of laser application for modification or processing of titanium dental implants. Lasers Med Sci. 2017;32(5):1197-206.

165. Davoudi A, Rismanchian M. Effects of modifying implant screw access channels on the amount of extruded excess cement and retention of cement-retained implant-supported dental prostheses: A systematic review. J Prosthet Dent. 2019;121(1):52-8.

166. Imani MM, Azizi F, Bahrami K, Golshah A, Safari-Faramani R. In vitro bleaching effect of hydrogen peroxide with different time of exposition and concentration on shear bond strength of orthodontic brackets to human enamel: A meta-analysis of in vitro studies. Int Orthod. 2020;18(1):22-31.

167. Samiei M, Shirazi S, Pournaghi Azar F, Fathifar Z, Ghojazadeh M, Alipour M. The Effect of Different Mixing Methods on the Properties of Calcium-enriched Mixture Cement: A Systematic Review of in Vitro Studies. Iranian Endodontic Journal. 2019;14(4):240-6.

168. Davoudi A, Mosharraf R, Akhavan A, Zarei F, Pourarz S, Iravani S. Effect of laser irradiation on push-out bond strength of dental fiber posts to composite resin core buildups: A systematic review and meta-analysis. Photodiagnosis Photodyn Ther. 2019;27:184-92.

169. Pourhajibagher M, Sodagar A, Bahador A. An in vitro evaluation of the effects of nanoparticles on shear bond strength and antimicrobial properties of orthodontic adhesives: A systematic review and meta-analysis study. Int Orthod. 2020;18(2):203-13.

170. Gorman CM, Ray NJ, Burke FM. The effect of endodontic access on all-ceramic crowns: A systematic review of in vitro studies. J Dent. 2016;53:22-9.

171. Tallarico M, Fiorellini J, Nakajima Y, Omori Y, Takahisa I, Canullo L. Mechanical Outcomes, Microleakage, and Marginal Accuracy at the Implant-Abutment Interface of Original versus Nonoriginal Implant Abutments: A Systematic Review of In Vitro Studies. Biomed Res Int. 2018;2018:2958982.

172. Savoldi F, Papoutsi A, Dianiskova S, Dalessandri D, Bonetti S, Tsoi JKH, et al. Resistance to sliding in orthodontics: misconception or method error? A systematic review and a proposal of a test protocol. Korean J Orthod. 2018;48(4):268-80.

173. Lombardo G, Pagano S, Cianetti S, Capobianco B, Orso M, Negri P, et al. Sub-ablative laser irradiation to prevent acid demineralisation of dental enamel. A systematic review of literature reporting in vitro studies. Eur J Paediatr Dent. 2019;20(4):295-301.

174. Corvino E, Pesce P, Mura R, Marcano E, Canullo L. Influence of Modified Titanium Abutment Surface on Peri-implant Soft Tissue Behavior: A Systematic Review of In Vitro Studies. Int J Oral Maxillofac Implants. 2020;35(3):503-19.

175. Masarwa N, Mohamed A, Abou-Rabii I, Abu Zaghlan R, Steier L. Longevity of Self-etch Dentin Bonding Adhesives Compared to Etch-and-rinse Dentin Bonding Adhesives: A Systematic Review. J Evid Based Dent Pract. 2016;16(2):96-106.

176. Heintze SD, Cavalleri A, Forjanic M, Zellweger G, Rousson V. Wear of ceramic and antagonist--a systematic evaluation of influencing factors in vitro. Dent Mater. 2008;24(4):433-49.

177. Dumbryte I, Vebriene J, Linkeviciene L, Malinauskas M. Enamel microcracks in the form of tooth damage during orthodontic debonding: a systematic review and meta-analysis of in vitro studies. Eur J Orthod. 2018;40(6):636-48.

178. Western JS, Dicksit DD. Apical extrusion of debris in four different endodontic instrumentation systems: A meta-analysis. J Conserv Dent. 2017;20(1):30-6.

179. Nagendrababu V, Jayaraman J, Suresh A, Kalyanasundaram S, Neelakantan P. Effectiveness of ultrasonically activated irrigation on root canal disinfection: a systematic review of in vitro studies. Clin Oral Investig. 2018;22(2):655-70.

180. Chia MSY, Parolia A, Lim BSH, Jayaraman J, Porto I. Effect of QMix irrigant in removal of smear layer in root canal system: a systematic review of in vitro studies. Restor Dent Endod. 2020;45(3):e28.

181. Cuevas-Suarez CE, da Rosa WLO, Lund RG, da Silva AF, Piva E. Bonding Performance of Universal Adhesives: An Updated Systematic Review and Meta-Analysis. The journal of adhesive dentistry. 2019;21(1):7-26.

182. Cuevas-Suarez CE, de Oliveira da Rosa WL, Vitti RP, da Silva AF, Piva E. Bonding Strength of Universal Adhesives to Indirect Substrates: A Meta-Analysis of in Vitro Studies. J Prosthodont. 2020;29(4):298-308.

183. Behring J, Junker R, Walboomers XF, Chessnut B, Jansen JA. Toward guided tissue and bone regeneration: morphology, attachment, proliferation, and migration of cells cultured on collagen barrier membranes. A systematic review. Odontology. 2008;96(1):1-11.

184. van Heumen CC, Kreulen CM, Bronkhorst EM, Lesaffre E, Creugers NH. Fiber-reinforced dental composites in beam testing. Dent Mater. 2008;24(11):1435-43.

185. Finnema KJ, Ozcan M, Post WJ, Ren Y, Dijkstra PU. In-vitro orthodontic bond strength testing: a systematic review and meta-analysis. Am J Orthod Dentofacial Orthop. 2010;137(5):615-22 e3.

186. Louropoulou A, Slot DE, Van der Weijden F. Influence of mechanical instruments on the biocompatibility of titanium dental implants surfaces: a systematic review. Clin Oral Implants Res. 2015;26(7):841-50.

187. Papageorgiou-Kyrana K, Fasoula M, Kontonasaki E. Translucency of Monolithic Zirconia after Hydrothermal Aging: A Review of In Vitro Studies. J Prosthodont. 2020;29(6):489-500.

188. Nilsen BW, Örtengren U, Simon-Santamaria J, Sørensen KK, Michelsen VB. Methods and terminology used in cell-culture studies of low-dose effects of matrix constituents of polymer resin-based dental materials. Eur J Oral Sci. 2016;124(6):511-25.

189. Możyńska J, Metlerski M, Lipski M, Nowicka A. Tooth Discoloration Induced by Different Calcium Silicate-based Cements: A Systematic Review of In Vitro Studies. J Endod. 2017;43(10):1593-601.

190. Kaczor K, Gerula-Szymanska A, Smektala T, Safranow K, Lewusz K, Nowicka A. Effects of different etching modes on the nanoleakage of universal adhesives: A systematic review and meta-analysis. J Esthet Restor Dent. 2018;30(4):287-98.

191. Gerula-Szymanska A, Kaczor K, Lewusz-Butkiewicz K, Nowicka A. Marginal integrity of flowable and packable bulk fill materials used for class II restorations -A systematic review and meta-analysis of in vitro studies. Dent Mater J. 2020;39(3):335-44.

192. Moreira AH, Rodrigues NF, Pinho AC, Fonseca JC, Vilaca JL. Accuracy Comparison of Implant Impression Techniques: A Systematic Review. Clin Implant Dent Relat Res. 2015;17 Suppl 2:e751-64.

193. Morado Pinho M, Manso MC, Martín C, Souza J, Almeida R, Pinhão Ferreira A. Adhesion strength of orthodontic brackets to acrylic surfaces. A systematic review on in vitro studies. Revista portuguesa de estomatologia e cirurgia maxilo-facial. 2017;58.

194. Al-Aali KA. Effect of phototherapy on shear bond strength of resin cements to zirconia ceramics: A systematic review and meta-analysis of in-vitro studies. Photodiagnosis Photodyn Ther. 2018;23:58-62.

195. AlFawaz YF, Alonaizan FA. Efficacy of phototherapy in the adhesive bonding of different dental posts to root dentin: A systematic review. Photodiagnosis Photodyn Ther. 2019;27:111-6.

196. Alamri A, Salloot Z, Alshaia A, Ibrahim MS. The Effect of Bioactive Glass-Enhanced Orthodontic Bonding Resins on Prevention of Demineralization: A Systematic Review. Molecules. 2020;25(11).

197. Astudillo-Rubio D, Delgado-Gaete A, Bellot-Arcis C, Montiel-Company JM, Pascual-Moscardo A, Almerich-Silla JM. Mechanical properties of provisional dental materials: A systematic review and meta-analysis. PLoS One. 2018;13(2):e0193162.

198. Amesti-Garaizabal A, Agustin-Panadero R, Verdejo-Sola B, Fons-Font A, Fernandez-Estevan L, Montiel-Company J, et al. Fracture Resistance of Partial Indirect Restorations Made With CAD/CAM Technology. A Systematic Review and Meta-analysis. J Clin Med. 2019;8(11).

199. Sanz JL, Forner L, Almudever A, Guerrero-Girones J, Llena C. Viability and Stimulation of Human Stem Cells from the Apical Papilla (hSCAPs) Induced by Silicate-Based Materials for Their Potential Use in Regenerative Endodontics: A Systematic Review. Materials (Basel). 2020;13(4).

200. Papia E, Larsson C, du Toit M, Vult von Steyern P. Bonding between oxide ceramics and adhesive cement systems: a systematic review. Journal of biomedical materials research Part B, Applied biomaterials. 2014;102(2):395-413.

201. Özcan M, Jonasch M. Effect of Cyclic Fatigue Tests on Aging and Their Translational Implications for Survival of All-Ceramic Tooth-Borne Single Crowns and Fixed Dental Prostheses. J Prosthodont. 2018;27(4):364-75.

202. Coray R, Zeltner M, Ozcan M. Fracture strength of implant abutments after fatigue testing: A systematic review and a meta-analysis. J Mech Behav Biomed Mater. 2016;62:333-46.

203. Baumgartner S, Koletsi D, Verna C, Eliades T. The Effect of Enamel Sandblasting on Enhancing Bond Strength of Orthodontic Brackets: A Systematic Review and Meta-analysis. The journal of adhesive dentistry. 2017;19(6):463-73.

204. Özcan M, Höhn J, Duarte Moura D, Souza R. Influence of testing parameters on the load-bearing capacity of prosthetic materials used for fixed dental prosthesis: A systematic review and meta-analysis. Brazilian Dental Science. 2018;21:470.

205. Yaylali IE, Kececi AD, Ureyen Kaya B. Ultrasonically Activated Irrigation to Remove Calcium Hydroxide from Apical Third of Human Root Canal System: A Systematic Review of In Vitro Studies. J Endod. 2015;41(10):1589-99.

206. Uzunoglu-Ozyurek E, Kucukkaya Eren S, Karahan S. Effect of root canal sealers on the fracture resistance of endodontically treated teeth: a systematic review of in vitro studies. Clin Oral Investig. 2018;22(7):2475-85.

207. Taha AA, Patel MP, Hill RG, Fleming PS. The effect of bioactive glasses on enamel remineralization: A systematic review. J Dent. 2017;67:9-17.

208. Lee H, So JS, Hochstedler JL, Ercoli C. The accuracy of implant impressions: a systematic review. J Prosthet Dent. 2008;100(4):285-91.

209. Kwon SR, Cortez E, Wang M, Jagwani M, Oyoyo U, Li Y. Systematic review of in vitro studies evaluating tooth bleaching efficacy. Am J Dent. 2020;33(1):17-24.

210. Deng K, Zhu C, Ma X, Jia H, Wei Z, Xiao Y, et al. Rapid Discrimination of Malignant Breast Lesions from Normal Tissues Utilizing Raman Spectroscopy System: A Systematic Review and Meta-Analysis of In Vitro Studies. PLoS One. 2016;11(7):e0159860.

211. Nuvvula S, Bhumireddy JR, Kamatham R, Mallineni SK. Diagnostic accuracy of direct digital radiography and conventional radiography for proximal caries detection in primary teeth: A systematic review. J Indian Soc Pedod Prev Dent. 2016;34(4):300-5.

212. Khalesi M, Jafari SA, Kiani M, Picarelli A, Borghini R, Sadeghi R, et al. In Vitro Gluten Challenge Test for Celiac Disease Diagnosis. J Pediatr Gastroenterol Nutr. 2016;62(2):276-83.

213. La Marca A, Capuzzo M, Paglia T, Roli L, Trenti T, Nelson SM. Testing for SARS-CoV-2 (COVID-19): a systematic review and clinical guide to molecular and serological in-vitro diagnostic assays. Reprod Biomed Online. 2020;41(3):483-99.

214. Oliveira PD, Pires-Oliveira DADA, Bertin LD, Szezerbaty SKF, Oliveira RF. The effect of therapeutic ultrasound on fibroblast cells in vitro: The systematic review. Archivos de Medicina del Deporte. 2018;35:50-5.

215. Rodrigues CdS, Aurélio IL, Kaizer MdR, Zhang Y, May LG. Do thermal treatments affect the mechanical behavior of porcelain-veneered zirconia? A systematic review and meta-analysis. Dental Materials. 2019;35(5):807-17.

216. Dias Corpa Tardelli J, Bolfarini C, Cândido dos Reis A. Comparative analysis of corrosion resistance between beta titanium and Ti-6Al-4V alloys: A systematic review. Journal of Trace Elements in Medicine and Biology. 2020;62:126618.

217. Goujat A, Abouelleil H, Colon P, Jeannin C, Pradelle N, Seux D, et al. Marginal and internal fit of CAD-CAM inlay/onlay restorations: A systematic review of in vitro studies. J Prosthet Dent. 2019;121(4):590-7 e3.

218. Ferle M, Guo R, Hurschler C. The Laxity of the Native Knee: A Meta-Analysis of in Vitro Studies. J Bone Joint Surg Am. 2019;101(12):1119-31.

219. Moharrami M, Perrotti V, Iaculli F, Love RM, Quaranta A. Effects of air abrasive decontamination on titanium surfaces: A systematic review of in vitro studies. Clin Implant Dent Relat Res. 2019;21(2):398-421.

220. Snijder RA, Konings MK, Lucas P, Egberts TC, Timmerman AD. Flow variability and its physical causes in infusion technology: a systematic review of in vitro measurement and modeling studies. Biomed Tech (Berl). 2015;60(4):277-300.

221. Boersema GS, Grotenhuis N, Bayon Y, Lange JF, Bastiaansen-Jenniskens YM. The Effect of Biomaterials Used for Tissue Regeneration Purposes on Polarization of Macrophages. BioResearch open access. 2016;5(1):6-14.

222. de Vries EE, Kok M, Hoving AM, Slump CH, Toorop RJ, de Borst GJ. (In)comparability of Carotid Artery Stent Characteristics: A Systematic Review on Assessment and Comparison with Manufacturer Data. Cardiovasc Intervent Radiol. 2020;43(10):1430-7.

223. Oliveira LB, Massignan C, Oenning AC, Rovaris K, Bolan M, Porporatti AL, et al. Validity of micro-CT for in vitro caries detection: a systematic review and meta-analysis. Dentomaxillofac Radiol. 2020;49(7):20190347.

224. Pintor AVB, Queiroz LD, Barcelos R, Primo LSG, Maia LC, Alves GG. MTT versus other cell viability assays to evaluate the biocompatibility of root canal filling materials: a systematic review. Int Endod J. 2020;53(10):1348-73.

225. Ellis JL, Alaiz-Moreton H, Navarro-Villa A, McGeough EJ, Purcell P, Powell CD, et al. Application of Meta-Analysis and Machine Learning Methods to the Prediction of Methane Production from In Vitro Mixed Ruminal Micro-Organism Fermentation. Animals (Basel). 2020;10(4).

226. Pisinger C, Godtfredsen N, Bender AM. A conflict of interest is strongly associated with tobacco industry-favourable results, indicating no harm of e-cigarettes. Preventive medicine. 2019;119:124-31.

227. Elshafay A, Omran ES, Abdelkhalek M, El-Badry MO, Eisa HG, Fala SY, et al. Reporting quality in systematic reviews of in vitro studies: a systematic review. Curr Med Res Opin. 2019;35(9):1631-41.

228. Sinha S, Sarma P, Sehgal R, Medhi B. Development in Assay Methods for in Vitro Antimalarial Drug Efficacy Testing: A Systematic Review. Frontiers in Pharmacology. 2017;8(754).

229. Salamanna F, Contartese D, Maglio M, Fini M. A systematic review on in vitro 3D bone metastases models: A new horizon to recapitulate the native clinical scenario? Oncotarget. 2016;7(28):44803-20.

230. Maglio M, Tschon M, Sicuro L, Lolli R, Fini M. Osteochondral tissue cultures: Between limits and sparks, the next step for advanced in vitro models. Journal of Cellular Physiology. 2018;234.

231. Kuik K, De Ruiter MHT, De Lange J, Hoekema A. Fixation methods in sagittal split ramus osteotomy: a systematic review on in vitro biomechanical assessments. Int J Oral Maxillofac Surg. 2019;48(1):56-70.

232. Hoving AM, de Vries EE, Mikhal J, de Borst GJ, Slump CH. A Systematic Review for the Design of In Vitro Flow Studies of the Carotid Artery Bifurcation. Cardiovasc Eng Technol. 2020;11(2):111-27.

233. Mai HY, Lee WK, Kwon TG, Lee DH. Reliability of digital measurement methods on the marginal fit of fixed prostheses: A systematic review and meta-analysis of in vitro studies. J Prosthet Dent. 2020;124(3):350 e1- e11.

234. Zhao S, Arnold M, Ma S, Abel RL, Cobb JP, Hansen U, et al. Standardizing compression testing for measuring the stiffness of human bone. Bone Joint Res. 2018;7(8):524-38.

235. Pasipanodya JG, Nuermberger E, Romero K, Hanna D, Gumbo T. Systematic Analysis of Hollow Fiber Model of Tuberculosis Experiments. Clin Infect Dis. 2015;61 Suppl 1:S10-7.

236. Jiang Z, He X, Li J. Synergy effect of meropenem-based combinations against Acinetobacter baumannii: a systematic review and meta-analysis. Infect Drug Resist. 2018;11:1083-95.

237. Bonczkowski P, De Scheerder MA, De Spiegelaere W, Vandekerckhove L. Minimal Requirements for Primary HIV Latency Models Based on a Systematic Review. AIDS Rev. 2016;18(4):171-83.

238. Tsouh Fokou PV, Nyarko AK, Appiah-Opong R, Tchokouaha Yamthe LR, Addo P, Asante IK, et al. Ethnopharmacological reports on anti-Buruli ulcer medicinal plants in three West African countries. J Ethnopharmacol. 2015;172:297-311.

239. Ilango KB, Kavimani S. A systematic review of mathematical models of pharmaceutical dosage forms. International Journal of Current Pharmaceutical Review and Research. 2015;6:59-70.

240. Baumeister D, Ciufolini S, Mondelli V. Effects of psychotropic drugs on inflammation: consequence or mediator of therapeutic effects in psychiatric treatment? Psychopharmacology (Berl). 2016;233(9):1575-89.

241. Ling C, An H, Li L, Wang J, Lu T, Wang H, et al. Genotoxicity Evaluation of Titanium Dioxide Nanoparticles In Vitro: a Systematic Review of the Literature and Meta-analysis. Biol Trace Elem Res. 2020.

242. Charles S, Jomini S, Fessard V, Bigorgne-Vizade E, Rousselle C, Michel C. Assessment of the in vitro genotoxicity of TiO2 nanoparticles in a regulatory context. Nanotoxicology. 2018;12(4):357-74.

243. Pronin S, Koh CH, Hughes M. Cytotoxicity of ultraviolet-C radiation on a heterogeneous population of human glioblastoma multiforme cells: Meta-analysis. Photodiagnosis Photodyn Ther. 2018;24:158-63.

244. Marigliani B, Sehn FP, Silva J, Balottin LBL, Augusto EFP, Buehler AM. The Overt and Hidden Use of Animal-Derived Products in Alternative Methods for Skin Sensitisation: A Systematic Review. Alternatives to laboratory animals : ATLA. 2019;47(5-6):174-95.
