# Supplementary material for: Quality assessment tools used in systematic reviews of in vitro studies: A systematic review
Source: BMC Med Res Methodol. 2021 May 8;21:101. doi: 10.1186/s12874-021-01295-w (PMC8106836; doi:10.1186/s12874-021-01295-w)
Supplement: Supplementary file 5 — Additional file 5: Table S5. Detailed result of DARE assessment criteria of included studies. [file 12874_2021_1295_MOESM5_ESM.docx]

**Table S5.** Detailed result of DARE assessment criteria of included studies.

| **ID** | **Author/publication year** | **Inclusion and exclusion** | **Search coverage** | **Assessment of quality** | **Study description** | **Synthesis of study** |
| --- | --- | --- | --- | --- | --- | --- |
| 1 | Wang/2020 | Y | P | Y | Y | N |
| 2 | Lenzi/2016 | Y | Y | Y | P | Y |
| 3 | Hasannejad-Bibalan/2019 | Y | P | Y | Y | Y |
| 4 | Pardo-Aldave/2019 | Y | Y | Y | Y | N |
| 5 | Diefenbach/2017 | Y | Y | Y | Y | N |
| 6 | Munitić/2019 | Y | P | Y | P | N |
| 7 | Pummer/2017 | Y | Y | N | Y | N |
| 8 | Alasqah/2019 | Y | P | N | Y | N |
| 9 | Lim/2020 | Y | P | Y | Y | N |
| 10 | Rêgo/2017 | Y | Y | Y | Y | N |
| 11 | Ellis/2020 | P | N | N | N | Y |
| 12 | Kirkegaard/2017 | N | P | Y | Y | N |
| 13 | Charles/2018 | Y | P | N | N | N |
| 14 | Swimberghe/2018 | Y | P | N | N | N |
| 15 | K/2017 | Y | Y | N | N | N |
| 16 | Jabran/2018 | Y | Y | N | N | N |
| 17 | Slette/2016 | Y | Y | N | N | N |
| 18 | Veronesi/2019 | P | P | N | N | N |
| 19 | Vasyutin/2019 | P | P | N | N | N |
| 20 | Thammajaruk/2018 | Y | Y | N | P | N |
| 21 | Cuevas-Suárez/2018 | Y | Y | Y | Y | Y |
| 22 | Cuevas-Suárez/2020 | Y | Y | Y | Y | Y |
| 23 | Yu/2019 | Y | P | Y | Y | Y |
| 24 | Soares/2016 | Y | P | Y | Y | Y |
| 25 | Mello/2018 | Y | Y | Y | Y | N |
| 26 | Bohrer/2018 | Y | P | Y | Y | Y |
| 27 | Oliveira/2018 | Y | P | Y | Y | N |
| 28 | Asweto/2017 | Y | Y | Y | Y | N |
| 29 | Chew/2020 | Y | P | N | N | N |
| 30 | Tardelli/2020 | Y | P | N | N | N |
| 31 | Pandey/2020 | Y | P | Y | Y | N |
| 32 | Herbst/2019 | Y | P | N | N | Y |
| 33 | Kaufmann/2018 | Y | Y | N | Y | N |
| 34 | Pisinger/2019 | P | P | N | N | N |
| 35 | Bryant/2018 | Y | Y | Y | Y | N |
| 36 | Tam/2020 | Y | Y | Y | Y | N |
| 37 | Pronin/2018 | P | N | N | Y | Y |
| 38 | Sinha/2017 | Y | P | N | N | N |
| 39 | Nuvvula/2016 | Y | P | Y | Y | N |
| 40 | Schestatsky/2018 | Y | P | Y | Y | Y |
| 41 | Razdan/2018 | Y | P | Y | P | N |
| 42 | Rodrigues/2019 | Y | P | Y | Y | Y |
| 43 | Al-Hamdan/2019 | Y | Y | N | Y | N |
| 44 | Nogueira/2020 | Y | P | Y | Y | N |
| 45 | Marchionatti/2018 | Y | Y | Y | P | Y |
| 46 | Brandeburski/2020 | Y | P | N | N | N |
| 47 | Fumes/2018 | Y | Y | N | Y | N |
| 48 | Miranda/2020 | Y | Y | Y | Y | N |
| 49 | Alamri/2020 | Y | P | Y | Y | N |
| 50 | Taha/2017 | Y | Y | Y | Y | N |
| 51 | Boersema/2016 | Y | Y | N | Y | N |
| 52 | Wehner/2020 | Y | P | Y | Y | Y |
| 53 | Picco/2019 | Y | Y | N | Y | N |
| 54 | Ozcan/2016 | Y | P | N | Y | N |
| 55 | Ren/2016 | Y | P | N | Y | N |
| 56 | Stefan/2017 | Y | Y | Y | Y | Y |
| 57 | Gorman/2016 | Y | Y | Y | Y | N |
| 58 | Mansourian/2020 | Y | P | N | Y | Y |
| 59 | Puidokas/2019 | Y | P | Y | Y | N |
| 60 | Holliday/2019 | Y | Y | Y | P | N |
| 61 | Kulkarni/2020 | Y | P | Y | Y | N |
| 62 | Al-Aali/2018 | Y | Y | N | Y | Y |
| 63 | Strauss/2019 | Y | P | N | Y | N |
| 64 | Elkaffas/2019 | Y | P | Y | Y | Y |
| 65 | Chia/2020 | Y | P | Y | Y | N |
| 66 | Uzunoglu-Özyürek/2018 | Y | Y | Y | Y | N |
| 67 | Mello/2017 | Y | P | N | Y | Y |
| 68 | Bangera/2020 | Y | Y | Y | Y | Y |
| 69 | Nagendrababu/2018 | Y | Y | Y | Y | N |
| 70 | Zhurakivska/2018 | Y | P | N | Y | N |
| 71 | Moharrami/2019 | Y | P | N | Y | N |
| 72 | Kaczor/2018 | Y | Y | Y | Y | Y |
| 73 | Napotnik/2016 | P | Y | Y | N | N |
| 74 | Scott/2019 | P | P | N | Y | N |
| 75 | Silva/2019 | Y | P | N | Y | N |
| 76 | Davoudi/2018 | Y | Y | Y | Y | N |
| 77 | Magrin/2020 | Y | Y | Y | Y | N |
| 78 | Almeida/2018 | Y | Y | Y | Y | Y |
| 79 | AlFawaz/2019 | Y | P | Y | Y | N |
| 80 | Parikh/2020 | Y | Y | Y | Y | N |
| 81 | Reis/2017 | Y | Y | Y | Y | N |
| 82 | Saravanan/2020 | Y | Y | N | N | N |
| 83 | Dumbryte/2018 | Y | Y | Y | Y | Y |
| 84 | Osmanovic/2018 | Y | P | Y | Y | N |
| 85 | Martins/2018 | Y | P | Y | Y | Y |
| 86 | Yu/2019 | Y | P | Y | Y | Y |
| 87 | Kuik/2018 | Y | P | Y | Y | N |
| 88 | Iliadi/2019 | Y | Y | Y | Y | Y |
| 89 | Mustafa/2020 | Y | P | N | Y | N |
| 90 | Amesti-Garaizabal/2019 | Y | Y | N | N | Y |
| 91 | Bethke/2020 | Y | P | N | Y | Y |
| 92 | Coraya/2016 | Y | P | N | Y | N |
| 93 | Ling/2020 | Y | Y | Y | Y | Y |
| 94 | Ferrúa/2017 | P | Y | Y | N | N |
| 95 | Martins/2018 | P | P | Y | Y | Y |
| 96 | Liu/2018 | Y | P | Y | N | Y |
| 97 | Rahman/2016 | Y | Y | Y | P | N |
| 98 | Silva/2017 | Y | Y | Y | Y | N |
| 99 | Bahsoun/2019 | Y | P | N | P | N |
| 100 | Li/2017 | Y | P | N | Y | Y |
| 101 | Maske/2017 | Y | P | Y | Y | N |
| 102 | Gianfredi/2017 | Y | P | N | N | N |
| 103 | Hindy/2017 | Y | P | Y | Y | N |
| 104 | Imani/2019 | Y | P | Y | Y | Y |
| 105 | Chemaly/2019 | Y | P | N | N | N |
| 106 | Caldas/2018 | Y | Y | Y | Y | N |
| 107 | Pacheco/2019 | Y | P | Y | Y | N |
| 108 | Mohammadrezaei/2018 | Y | P | N | Y | N |
| 109 | Resende/2019 | Y | Y | Y | Y | N |
| 110 | Pourhajibagher/2020 | Y | P | Y | Y | Y |
| 111 | Khalesi/2016 | Y | P | Y | Y | Y |
| 112 | Hoppenbrouwers/2017 | P | P | N | P | N |
| 113 | Hellwig/2019 | Y | P | N | P | N |
| 114 | Sousa/2018 | Y | Y | Y | Y | N |
| 115 | Janjic/2018 | Y | P | N | P | N |
| 116 | Vries/2020 | Y | P | N | P | N |
| 117 | Leão/2020 | Y | P | Y | Y | N |
| 118 | Perroni/2018 | Y | Y | Y | Y | N |
| 119 | Corvino/2020 | Y | Y | Y | Y | N |
| 120 | Kreve/2020 | Y | Y | N | Y | N |
| 121 | Rosa/2016 | Y | Y | Y | Y | Y |
| 122 | Nawafleh/2016 | Y | P | N | P | N |
| 123 | Masarwa/2016 | Y | Y | Y | Y | Y |
| 124 | Goujat/2019 | Y | Y | Y | Y | N |
| 125 | Gerula-Szymańska/2020 | Y | P | Y | Y | Y |
| 126 | Menezes-Silva/2018 | Y | P | Y | Y | N |
| 127 | Tallarico/2018 | Y | P | Y | Y | N |
| 128 | Astudillo-Rubio/2018 | Y | P | Y | Y | Y |
| 129 | Lemma/2017 | Y | Y | N | P | N |
| 130 | Maccarana/2016 | Y | P | N | Y | P |
| 131 | Nilsen/2016 | Y | Y | N | Y | N |
| 132 | Silveira/2020 | Y | Y | Y | Y | N |
| 133 | Pintor/2020 | Y | P | Y | Y | P |
| 134 | Shahmiri/2017 | Y | Y | N | P | N |
| 135 | Cury/2019 | Y | Y | Y | Y | N |
| 136 | Maglio/2018 | Y | P | N | Y | N |
| 137 | Marigliani/2020 | Y | Y | N | P | N |
| 138 | Münchow/2018 | Y | Y | Y | N | Y |
| 139 | Pardal-Peláez/2017 | Y | P | N | Y | N |
| 140 | Deng/2016 | Y | P | Y | Y | Y |
| 141 | Mai/2020 | Y | Y | Y | Y | Y |
| 142 | Elshafay/2019 | Y | Y | Y | Y | P |
| 143 | Savoldia/2018 | P | P | N | Y | N |
| 144 | Dissemond/2020 | Y | P | N | P | N |
| 145 | Jayanegara/2018 | Y | N | N | Y | P |
| 146 | Zhao/2018 | Y | Y | N | N | N |
| 147 | Lombardo/2019 | Y | P | N | N | Y |
| 148 | Gianfredi/2017 | Y | P | N | Y | Y |
| 149 | Khanafer/2017 | Y | N | N | N | Y |
| 150 | Jiang/2018 | Y | P | N | Y | Y |
| 151 | Hoving/2019 | Y | Y | N | Y | N |
| 152 | Hlashwayo/2020 | Y | Y | N | Y | N |
| 153 | Daltona/2019 | Y | P | N | Y | N |
| 154 | Kwon/2020 | Y | P | Y | Y | Y |
| 155 | Prpa/2020 | Y | Y | Y | P | N |
| 156 | Khaledi/2020 | Y | Y | Y | Y | N |
| 157 | Almoudi/2018 | Y | P | N | P | N |
| 158 | Marca/2020 | P | P | N | Y | N |
| 159 | Mozynska/2017 | P | P | Y | Y | N |
| 160 | Franzoni/2017 | P | N | P | P | N |
| 161 | Papageorgiou-Kyrana/2020 | Y | P | N | Y | N |
| 162 | Lemos/2017 | Y | P | N | Y | N |
| 163 | Chierrito/2019 | Y | P | N | Y | N |
| 164 | Oliveira/2019 | Y | Y | Y | Y | Y |
| 165 | Sanz/2020 | Y | Y | Y | Y | N |
| 166 | Carvalho/2018 | Y | Y | Y | Y | Y |
| 167 | Ferle/2019 | P | P | N | Y | Y |
| 168 | Pires/2018 | Y | P | Y | Y | Y |
| 169 | Western/2017 | Y | P | Y | P | Y |
| 170 | Solanki/2018 | Y | P | N | N | N |
| 171 | Li/2019 | Y | P | Y | N | N |
| 172 | Fonseca/2020 | Y | P | Y | Y | P |
| 173 | Ajay/2019 | Y | P | Y | Y | Y |
| 174 | Moradi/2016 | Y | Y | N | P | N |
| 175 | Oliveira/2018 | Y | Y | N | Y | Y |
| 176 | Pinho/2017 | Y | Y | Y | Y | Y |
| 177 | Elshiyab/2017 | Y | Y | Y | Y | Y |
| 178 | Samiei/2019 | Y | Y | Y | Y | Y |
| 179 | Abreu/2019 | Y | Y | Y | Y | Y |
| 180 | Ozcan/2018 | Y | Y | Y | Y | Y |
| 181 | Tavares/2019 | Y | Y | Y | Y | Y |
| 182 | Davoudi/2019 | Y | Y | Y | Y | Y |
| 183 | Tan/2018 | Y | Y | Y | Y | Y |
| 184 | Correa/2018 | Y | Y | Y | Y | Y |
| 185 | Garcia-Sanz/2018 | Y | Y | Y | Y | Y |
| 186 | Sanches/2020 | Y | Y | Y | Y | Y |
| 187 | Gentile/2020 | Y | Y | Y | P | N |
| 188 | Gizani/2020 | Y | P | Y | Y | N |
| 189 | Kumar/2016 | Y | P | N | P | N |
| 190 | Moreira/2015 | Y | P | N | Y | Y |
| 191 | Lee/2008 | Y | Y | N | Y | Y |
| 192 | Gizzo/2015 | Y | Y | N | Y | P |
| 193 | Altmann/2016 | P | Y | Y | Y | Y |
| 194 | Bates/2015 | Y | Y | N | Y | Y |
| 195 | Tong/2015 | P | Y | N | Y | Y |
| 196 | Pereiraa/2015 | Y | Y | Y | Y | Y |
| 197 | Pasipanodya/2015 | P | Y | N | Y | Y |
| 198 | Ilango/2015 | N | N | N | Y | Y |
| 199 | Rosa/2015 | P | Y | Y | Y | Y |
| 200 | Papia/2014 | Y | P | N | Y | Y |
| 201 | Moraes/2015 | Y | P | Y | Y | Y |
| 202 | Chaves/2012 | Y | Y | N | Y | Y |
| 203 | Nassar/2011 | Y | Y | N | Y | Y |
| 204 | Kaizer/2014 | P | Y | N | Y | Y |
| 205 | Aurelio/2016 | Y | Y | Y | Y | Y |
| 206 | Shahravan/2007 | Y | P | N | Y | Y |
| 207 | Bleuel/2015 | Y | P | N | Y | Y |
| 208 | Baumeister/2016 | Y | Y | N | Y | Y |
| 209 | Fokou/2015 | Y | Y | N | Y | Y |
| 210 | Arilla/2015 | Y | Y | Y | Y | Y |
| 211 | Heumen/2008 | Y | P | N | Y | Y |
| 212 | Snijder/2015 | Y | P | N | Y | Y |
| 213 | Ehsani/2009 | Y | Y | Y | Y | Y |
| 214 | Alshwaimi/2016 | Y | Y | Y | Y | Y |
| 215 | Pavan/2015 | Y | Y | Y | Y | Y |
| 216 | Ni/2015 | Y | Y | N | Y | Y |
| 217 | Passos/2014 | Y | Y | Y | Y | Y |
| 218 | Bernades/2014 | Y | P | N | Y | Y |
| 219 | Louropoulou/2015 | Y | Y | Y | Y | Y |
| 220 | Rotelli/2015 | Y | Y | N | P | Y |
| 221 | Finnema/2010 | Y | P | N | Y | Y |
| 222 | Tzanakakis/2016 | Y | P | N | Y | Y |
| 223 | Xiao/2011 | Y | Y | P | Y | Y |
| 224 | Montano/2010 | Y | Y | N | Y | Y |
| 225 | Costa/2013 | Y | Y | N | Y | Y |
| 226 | Jayanegara/2014 | Y | Y | N | Y | Y |
| 227 | Schmid-Schwap/2011 | Y | Y | N | Y | Y |
| 228 | Bonczkowski/2016 | Y | P | N | Y | Y |
| 229 | Samiei/2016 | Y | Y | P | Y | Y |
| 230 | Dobbenga/2016 | Y | Y | N | Y | Y |
| 231 | Peplow/2013 | Y | P | N | Y | Y |
| 232 | Sarkis-Onofre/2014 | Y | P | Y | Y | Y |
| 233 | Motamedian/2015 | Y | P | N | Y | Y |
| 234 | Zusman/2013 | Y | Y | N | Y | Y |
| 235 | Skupien/2015 | Y | P | N | Y | Y |
| 236 | Tabatabaei-Malazy/2012 | Y | Y | N | Y | Y |
| 237 | Ting/2016 | Y | Y | N | Y | Y |
| 238 | Salamanna/2016 | Y | Y | N | Y | Y |
| 239 | Archambault/2010 | Y | Y | N | Y | Y |
| 240 | Behring/2008 | P | N | N | Y | Y |
| 241 | Contreras-Ochoa/2012 | P | Y | N | Y | Y |
| 242 | Yaylali/2015 | Y | Y | Y | Y | Y |
| 243 | Heintze/2008 | Y | P | N | Y | Y |
| 244 | Golbach/2016 | Y | Y | Y | Y | Y |

Y: Yes; P: Partial; N: No.
