# Supplementary figures and images for: Quality assessment tools used in systematic reviews of in vitro studies: A systematic review
Source: BMC Med Res Methodol. 2021 May 8;21:101. doi: 10.1186/s12874-021-01295-w (PMC8106836; doi:10.1186/s12874-021-01295-w)

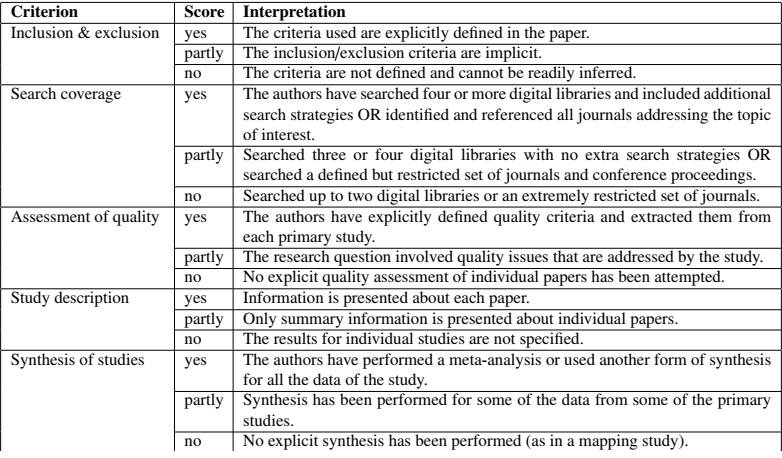

Supplement: Supplementary file 6 — Additional file 6: Figure S1. The interpretation for fulfilling a “yes”, “partial” and “no” score. [file 12874_2021_1295_MOESM6_ESM.jpg]
